# Intrinsically stretchable organic light-emitting-diode with high brightness and stretchability via elastic-microphase-engineered emitter and dual-embedded electrode

**DOI:** 10.1038/s41377-026-02271-z

**Published:** 2026-03-25

**Authors:** Zhen Lu, Jiaming Huang, Qiong Liang, Kuan Liu, Dongyang Li, Jiayuan Zhu, Cenqi Yan, Yaokang Zhang, Heng Liu, Xinhui Lu, Jianjun Tian, Zi Jing Wong, Gang Li

**Affiliations:** 1 Department of Electrical and Electronic Engineering, Research Institute for Smart Energy (RISE); Photonic Research Institute (PRI), The Hong Kong Polytechnic University, Hung Hom, Kowloon. Hong Kong China; 2 School of Electronic Science and Technology, Eastern Institute of Technology, Ningbo, 315200 China; 3 College of Polymer Science and Engineering, State Key Laboratory of Polymer Materials Engineering, Sichuan University, Chengdu, 610040 China; 4 College of Chemistry and Environmental Engineering, Shenzhen University, Shenzhen, 518055 China; 5 Department of Physics, The Chinese University of Hong Kong, Shatin, Hong Kong China; 6 Institute for Advanced Materials and Technology, University of Science and Technology Beijing, 100083 Beijing, China; 7 Guangdong-Hong Kong-Macao Joint Laboratory for Photonic-Thermal-Electrical Energy Materials and Devices, The Hong Kong Polytechnic University, Hung Hom, Kowloon. Hong Kong China

**Keywords:** Organic LEDs, Photonic devices

## Abstract

Intrinsically stretchable organic light-emitting diodes (is-OLEDs), composed entirely of inherently stretchable functional layers, represent a promising enabling technology for wearable electronics due to stretching and deformation endurance during the dynamic movements of the human body. However, achieving high performance and stretchability is challenging due to limited ductility in conjugated emissive materials and the low quality of stretchable transparent electrodes (STEs). In this study, we explore an elastic-microphase-engineered emissive layer strategy for is-OLEDs. This involves investigating the effects of incorporating different styrene-butadiene-styrene block copolymer derivative elastomers into a green polyfluorene emissive polymer. The miscibility between elastomers and emissive polymers is found to be critical in regulating three-dimensional microphase separation in the blend, thus simultaneously affecting mechanical and optoelectronic properties. In addition, by pre-burying conductive polymer PH1000, the smoothed dual-embedded hybrid electrode PH1000@AgNWs@TPU (PAT) STE with superior conductivity, stretchability, and stability is achieved. As a result, the obtained is-OLED demonstrated a record luminance of 33,443 cd m^−2^ and stretchability up to 120%, while also maintaining approximately 90% of its initial luminance after 100 cycles of dynamic stretching at 15% strain, representing a significant stride towards realizing the full potential of is-OLEDs for next-generation wearable electronic applications.

## Abstract (web summary)

Elastic-microphase-engineering and dual-embedded electrodes enable intrinsically stretchable OLEDs with record brightness (33,443 cd/m²) and stretchability (120%).

## Graphical abstract

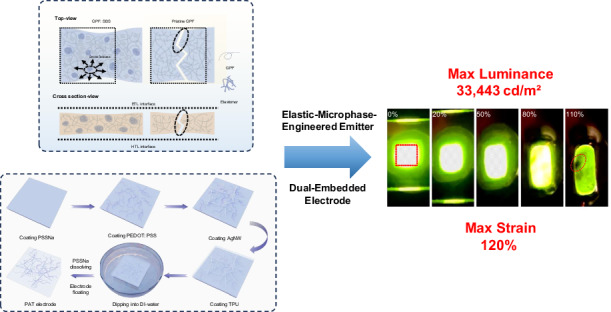

## Introduction

Organic light-emitting diodes (OLEDs) have become essential components in display technology due to high contrast, high brightness, wide viewing angles, light-weight, color-tunability, and flexibility.^1–7^ For wearable smart devices,^8^ conventional OLEDs, including both rigid and flexible types,^9–11^ face challenges in operating under large strain of 40–100% in different human-body parts.^12,13^ Intrinsically stretchable OLEDs (*is*-OLEDs), characterized by all constituent functional layers being comprised of stretchable materials, represent a promising alternative solution for wearable applications.^14–30^ To realize intrinsically stretchable OLEDs, recent important advances such as designing novel thermally activated delayed fluorescence (TADF) polymers with both high ductility and efficiency,^17,31^ as well as introducing fluid conjugated molecular external-plasticizing^19^ were reported. In addition, device engineering approaches, such as employing cross-linkable layers to secure interface morphology in multilayer stacks, have also proven effective for fabrication stability.^32^ Besides, physical blending with elastomers stands out as a particularly versatile strategy, utilizing a soft matrix to dissipate strain while preserving optoelectronic properties. On the other hand, conventional transparent electrode materials, such as indium tin oxide (ITO) and ultra-thin metals, are prone to cracking under tensile strain.^33–37^ So far, no is-OLED has been reported to simultaneously achieve a stretchability exceeding 100%, a luminance surpassing 10,000 cd m^−^, and an external quantum efficiency (EQE) over 2%.

Doping elastomers into the emissive material is a commonly used strategy to enhance stretchability.^16,22,23,38–40^ However, insulating elastomers would impede charge transport within the emissive layer thus reducing efficiency. While certain reports indicate that specific elastomers can enhance the electrical performance of semiconductors,^16,41,42^ achieving an optimal equilibrium between electroluminescence performance and stretchability requires meticulously selected combinations. Furthermore, the lack of guidelines for dopant selection adds complexity to this process.

Equally important, *is*-OLEDs require high-quality stretchable transparent electrodes (STEs). Silver nanowires (AgNWs) stand out as promising candidates instead of ITO, due to their excellent conductivity, high transparency, and ability to accommodate mechanical strain while preserving network integrity.^43–45^ Despite these advantages, AgNW-based electrodes face issues such as junction resistance, large surface roughness, limited adhesion to underlying substrates, and poor stability.^15,37,46–48^ These issues can lead to network fractures or delamination, compromising device stability and efficiency.

Herein, we proposed an elastic-microphase-engineering approach that simultaneously achieves high optoelectronic and mechanical performance. We incorporate three elastomers with varying stretchability: styrene-butadiene-styrene (SBS), styrene-isoprene-styrene (SIS), and styrene-ethylene-butylene-styrene (SEBS) blend with a green polyfluorene emissive polymer^49,50^ (GPF). Our investigation reveals that the GPF:SBS composition, driven by superior thermodynamic miscibility, self-assembles into a unique three-dimensional phase morphology. Within this structure, GPF serves as the continuous host matrix, providing charge transport channels and emissive functionality, while SBS is uniformly dispersed throughout the three-dimensional structure to act as nanoscale tensile-dissipation sites and effectively reduce electron traps. This stands in sharp contrast to the SIS and SEBS blends, which suffer from severe phase separation in both lateral and vertical dimensions due to surface energy mismatch. On one hand, vertical phase separation impedes interfacial charge injection, causing carrier imbalance. On the other hand, the uneven distribution creates stress concentration, resulting in premature localized fracture. The optimal three-dimensional phase separation through Elastic-Microphase-Engineering effectively resolves the trade-off between electrical conductivity and mechanical robustness. As a result, the fabricated is-OLED simultaneously exhibits superior brightness, efficiency, and stretchability.

Subsequently, a hybrid STE, denoted as PH1000@AgNWs@TPU (PAT), which incorporates a pre-buried PH1000 layer and a sacrificial poly (sodium 4-styrenesulfonate) (PSSNa) layer, was designed. Benefitting from the dual-embedded structure, PAT STE exhibited higher conductivity, adhesion, stretchability, and long-term stability. Based on the GPF: 10%SBS emissive layer and PAT STE, we demonstrate *is*-OLED with record performance (brightness of 33,443 cd m^−2^, EQE of 2.3%, and current efficiency of nearly 8 cd A^–1^). Moreover, the *is*-OLED exhibited excellent stretchability of up to 120% and demonstrated excellent stability by retaining approximately 90% of its initial luminance after 100 stretching and releasing cycles at 15% strain. This work presents promising strategies for achieving a combination of exceptional mechanical stretchability and outstanding optoelectronic performance, paving the way for the future development of *is*-OLED.

## Results

### Mechanical stretchability

**Fig. 1:**
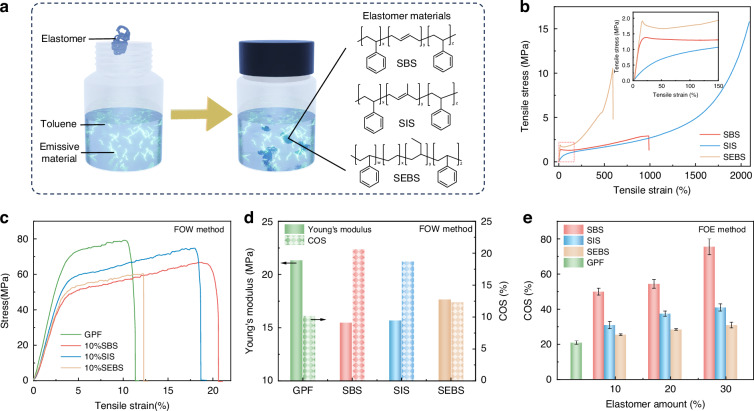
Chemical structure and mechanical properties of elastomers and GPF-based blend films. **a** Schematic illustration of an emissive material mixed with elastomers in solution and chemical structure of the three elastomers: SBS, SIS, and SEBS. **b** Stress-strain curves of SBS, SIS, and SEBS. **c** Tensile stress curves of FOW for pristine GPF and GPF blended with 10% SBS, SIS, or SEBS (weight ratios). **d** Young’s modulus and COS (obtained from FOW) values of pristine GPF and GPF blended with 10% SBS, SIS, or SEBS (weight ratios). **e** COS of pristine GPF and GPF blended with 10%, 20%, and 30% (weight ratios) of each elastomer in the FOE method

The intrinsically stretchable emissive layer is a crucial component in *is*-OLEDs. The incorporation of soft elastomers could enhance the stretchability of blended films by effectively reducing their overall modulus (Fig. 1a). Three elastomers (SBS, SIS, SEBS) (Fig. 1a) with similar chemical structure but different stretchability and surface property were selected for blending with the emissive material GPF. All three elastomers demonstrate excellent ductility, yet their mechanical properties exhibit differences. Figure 1b shows the stress-strain curves for three elastomers. The crack-on-set (COS) values are 990% for SBS, 2090% for SIS, and 600% for SEBS. Their Young’s moduli are 13.7 MPa, 2.2 MPa, and 14.0 MPa, respectively. SIS exhibits the highest stretchability, followed by SBS, while SEBS demonstrates the lowest value. The mechanical properties of GPF: elastomer blended films were then evaluated. Tensile stress-strain measurements were performed using the film-on-water^51^ (FOW) method (Fig. 1c, Supplementary Fig. 1), demonstrating that the GPF: SBS blended film showed the greatest enhancement in mechanical properties. Young’s modulus and crack-on-set (COS) values were calculated (Fig. 1d). Among the blends, SBS incorporation resulted in the lowest Young’s modulus: 2134 MPa for pristine GPF, 1547 MPa for GPF:10%SBS, 1567 MPa for GPF:10%SIS, and 1763 MPa for GPF:10%SEBS. The measured COS values were 10.18%, 20.65%, 18.67%, and 12.29%, respectively. SBS incorporation significantly increased the COS, achieving a value twice that of the pristine GPF film. SIS showed the second highest COS improvement, while SEBS only slightly increased the COS. The tensile behavior was further examined using the film-on-elastomer (FOE) method (Fig. 1e, Supplementary Fig. 2–5) to evaluate stretchability under practical conditions. While FOW quantifies the intrinsic mechanical parameters in a quasi-free-standing state, FOE assesses the practical stretchability of the film when supported by a substrate, simulating the actual mechanical environment within the device. Blended films with 10% SBS showed a 238% increase in COS compared to pristine GPF. A 10% SIS blend film provided a 148% improvement in COS, whereas a 10% SEBS blend film showed lower values (121%) than both SBS and SIS. Differential scanning calorimetry (DSC) measurements (Supplementary Fig. 6) revealed a reduced glass transition temperature (T_g_) for 10% SBS and 10% SIS blends, confirming enhanced chain mobility consistent with their improved stretchability, whereas the 10%SEBS blend showed an increased T_g_. Supplementary Fig. 7 demonstrates the maintained photoluminescence of the GPF: 10% SBS blend film even when stretched from 0% to 440%, and the uniform emitting region even after a large mechanical strain.

The stretchability ranking of blended films differs from that of the pristine elastomers. Specifically, the blend film with SIS, the most stretchable elastomer, shows lower stretchability compared to the blend film with SBS. To investigate this, we examined the miscibility of the elastomers and GPF. Poor miscibility can lead to large phase separation, which in turn affects the mechanical properties of the blend film. We calculated the Flory-Huggins interaction parameter χ (Supplementary Table 1) by using contact angle (Supplementary Fig. 8) measurements to assess miscibility. A smaller interaction parameter indicates improved miscibility, reducing the propensity for phase separation. SBS exhibited contact angles of 99.64° with water and 70.72° with ethylene glycol, resulting in a surface energy of 27.40 mN m^–1^, closely matching that of GPF (27.14 mN m^–1^). The interaction parameter between SBS and GPF was the lowest (χ_GPF-SBS_ = 6.6 × 10^–4^ K), indicating excellent miscibility at the molecular level. Conversely, SEBS demonstrated the poorest miscibility with GPF. Its contact angles were 101.18° (water) and 72.29° (ethylene glycol), yielding a higher surface energy of 32.8 mN m^–1^. Moreover, SEBS had the highest interaction parameter with GPF (χ_GPF-SEBS_ = 2.71 × 10^–1^ K), suggesting a strong propensity for phase separation. SIS exhibited intermediate miscibility, demonstrating a moderate interaction parameter with GPF (χ_GPF-SIS_ = 4.7 × 10^–2^ K). While SIS may blend with GPF to some extent, this interaction is insufficient to prevent microphase separation. We concluded that component miscibility is a more critical factor in determining the stretchability of the blended film than the modulus of the elastomers. In a blend film, poor miscibility between components can cause regions with varying mechanical properties to form. Under stress, regions with lower elastomer content may fracture prematurely, which results from uneven elastomer distribution. This localized failure compromises the overall mechanical performance of the film.

### Optoelectronic performances

**Fig. 2:**
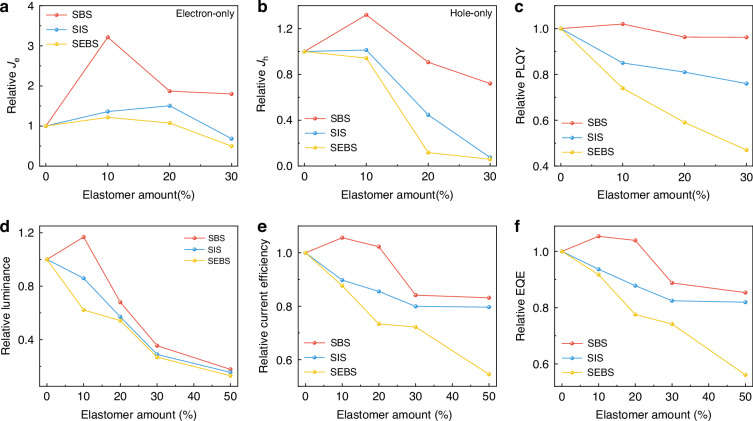
Effects of elastomer content on optoelectronic properties of GPF-based blend films. **a** Relative electron current densities, **b** relative hole current densities, **c** relative PLQY, **d** relative luminance, **e** relative current efficiency, **f** relative EQE in GPF: Elastomers thin films as the elastomer content (weight ratios) increases

The effect of elastomer dopants on the optoelectronic performance of GPF was then investigated. The transmittance of each elastomer was evaluated within the GPF emission range, which all exhibited over 98% transmittance (Supplementary Fig. 9). Absorption spectra of the blended films with three elastomers were consistent with that of pristine GPF (Supplementary Fig. 10). Electron-only and hole-only devices were fabricated to monitor electron and hole currents, respectively, in order to examine how elastomer addition affected charge transport characteristics (Fig. 2a, b, Supplementary Figs. 11-12). As expected, the electron current density in pristine GPF was approximately three orders of magnitude lower than the hole current density, primarily due to the electron current being strongly trap-limited.^52^ With 10% dopant, the SBS-based blend film showed a significant increase in electron current density, rising from 0.12 mA cm^−2^ to 0.38 mA cm^−2^ at 8 V, a greater than 300% enhancement. 10% SEBS-based blend showed limited improvement in current density, likely due to the negative impact of poor miscibility and phase separation,^41^ while the SIS-based film blend showed moderate gains in charge transport. However, both electron and hole current densities decreased at higher elastomer concentrations, due to a decreased ratio of emissive material and disrupted charge transport pathways.

Photoluminescent quantum yield (PLQY) measurements (Fig. 2c, Supplementary Fig. 13) showed that incorporating 10% SBS slightly enhances the blended film’s PLQY, which remains near the pristine GPF level with increasing concentration. In contrast, SEBS and SIS significantly decrease the blended film’s PLQY. Time-resolved photoluminescence (TRPL) measurements (Supplementary Fig. 14) support these findings, revealing the largest increase in exciton lifetime (Supplementary Table 2) for the 10% SBS blend (from 1.17 to 1.71 ns), indicating reduced non-radiative decay and improved exciton stability, consistent with electron and hole current data. Notably, a clear blue shift in the emission of SEBS- and SIS-based blends was observed, attributed to decreased crystallinity and increased molecular disorder in the blends (Supplementary Fig. 15). In contrast, the SBS: GPF blends exhibited a narrower full width at half maximum (FWHM) with no significant peak shift, indicating that the incorporation of SBS positively impacts the molecular order within the blends.

We selected the 10% doping concentration for detailed analysis. This ratio yields the peak optoelectronic output. At higher concentrations, the insulating elastomer significantly disrupts charge transport networks. To evaluate the optoelectronic performance of OLED, we fabricated devices with the structure ITO/PEDOT: PSS/blended films/PFNBr: PEIE/Ag (Supplementary Figs. 16–19, Supplementary Table 3). Specifically, at a 10% incorporation level, SBS blended films showed the highest luminescence of 39981 cd m^–2^. Concurrently, these films also exhibited the highest current efficiency of 7.52 cd A^–1^ and external quantum efficiency (EQE) of 2.16%. The peak luminance was approximately 1.17 times that of pristine GPF (Fig. 2d). Furthermore, the current efficiency and EQE increased by about 1.06 times (Fig. 2e, f). Blending the emissive material with an appropriate amount of elastomer reduces trap-limited electron transport, thereby enabling a balanced transport of positive and negative charges to enhance device efficiency.^41,53^ Collectively, these findings demonstrate that SBS is the most effective elastomer for improving both the mechanical and optoelectronic properties of the emissive layer, whereas SIS offers only a limited improvement. SEBS is unsuitable for this application due to its severe phase separation and poor miscibility.

### Morphology evolution

**Fig. 3:**
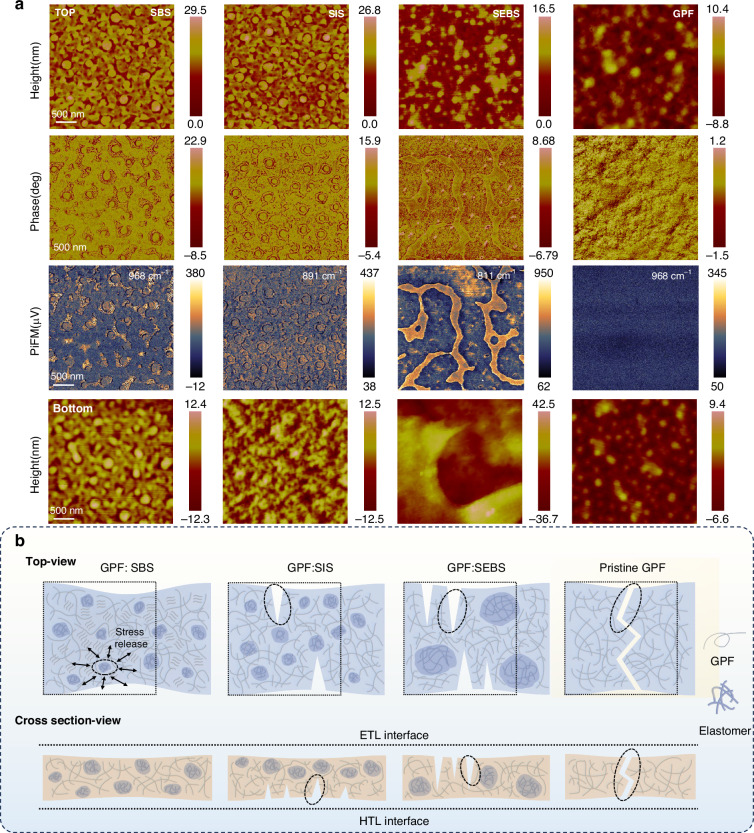
Morphological properties of GPF-based blend films. **a** AFM top height and phase images, bottom height images, and PiFM images of pristine GPF, GPF: 10%SBS, GPF: 10%SIS, and GPF: 10%SEBS films. The surface roughness parameters, Root Mean Square roughness (Rq) and Maximum peak-to-valley height (Rmax), derived from the top height images are: GPF:10%SBS (Rq = 2.798 nm, Rmax = 46.05 nm), GPF:10%SIS (Rq = 2.677 nm, Rmax = 28.04 nm), GPF:10%SEBS (Rq = 2.235 nm, Rmax = 17.84 nm), and pristine GPF (Rq = 2.80 nm, Rmax = 18.2 nm). Roughness parameters derived from the bottom height images are: GPF:10%SBS (Rq = 3.16 nm, Rmax = 25.8 nm), GPF:10%SIS (Rq = 3.58 nm, Rmax = 24.9 nm), GPF:10%SEBS (Rq = 8.59 nm, Rmax = 70.2 nm), and pristine GPF (Rq = 2.45 nm, Rmax = 15.9 nm). **b** A schematic illustration of the mechanism for improving optoelectronic and stretchable properties. Top-view: This depicts the lateral microphase distribution. The dashed square represents the initial, unstretched state. Dashed circles indicate fracture-induced cracks. Cross sectional-view: This illustrates the vertical microphase distribution

The miscibility between GPF and elastomers would impact the morphology of blend films. Atomic force microscopy (AFM), photo-induced Force Microscopy (PiFM) and transmission electron microscopy (TEM) was used to gain a deeper understanding of the morphology, specifically the phase separation, of the blended films and its influence on device performance (Fig. 3a, Supplementary Figs. 20–23). The pristine GPF film exhibited a mostly uniform surface, with the height image displaying slight variations in height and the phase image showing a smooth, consistent texture. In contrast, the GPF: SEBS blend shows significant phase separation. The SEBS phase forms large, interconnected regions, which appear quite distinct from the GPF. Both the height and phase images demonstrate this phase separation. This indicates a lack of miscibility between GPF and SEBS, which shows a strong degree of segregation. The GPF: SBS and GPF: SIS blends exhibit a more favorable morphology. The SBS and SIS phases appear as generally uniform, rounded domains. These domains are dispersed in a relatively regular manner throughout the GPF. They are more consistent in size and distribution compared to the SEBS blend. This difference in morphology is consistent with the observed variations in mechanical properties. The SEBS blend exhibits the poorest performance due to its uneven distribution, which leads to localized stress concentrations and premature fracture in regions deficient in SEBS. Additionally, in the GPF: SBS blend, many small particles are observed, which have an ordered arrangement within the GPF. These particles are distinct from the SBS domains and could be suggestive of induced crystallization of the GPF phase in the presence of the SBS elastomer.

In addition to top-surface AFM, we also characterized the bottom surface of the blended films (Fig. 3a). This analysis revealed distinct vertical distributions of the elastomers. For the GPF:SBS blend, SBS nanodomains were still observable on the bottom surface. In contrast, SIS was nearly absent from the bottom surface of the GPF:SIS blend. Conversely, the GPF:SEBS blend showed an enrichment of SEBS at its bottom interface compared to its top surface. These observations can be attributed to interfacial energetics, particularly the high surface energy of the underlying PEDOT:PSS substrate^54^ (typically ˃40 mN m^–1^). SIS possesses a low surface energy (24.9 mN m^–1^), significantly lower than that of PEDOT:PSS. Consequently, SIS preferentially migrated towards the upper, lower-energy interface, away from the high-energy PEDOT:PSS substrate. SEBS exhibits a surface energy of 32.8 mN m^–1^. This value is higher than that of SBS, SIS, and GPF, and it is the closest to the surface energy of PEDOT:PSS. However, due to its exceptionally poor miscibility with GPF, SEBS tends to form large, segregated domains. These domains preferentially accumulate near the bottom interface, adjacent to the PEDOT:PSS substrate. The SBS elastomer has a surface energy of 27.4 mN m^–1^. While this is also lower than that of PEDOT:PSS. The excellent miscibility with GPF of SBS promotes a more uniform distribution throughout the film. As a result, SBS maintained a relatively uniform vertical distribution. The lateral and vertical microphase distributions collectively establish the overall three-dimensional morphology of the blended film. In contrast, poor miscibility between components has detrimental effects on this three-dimensional structure. It not only induces large-scale phase separation in the lateral plane but can also cause increased phase separation in the vertical direction, leading to serious injection barriers. By carefully tuning component miscibility, our ‘Elastic-Microphase Engineering’ aim to achieve a uniformly distributed blend in both lateral and vertical dimensions, thereby enabling the simultaneous enhancement of stretchability and optoelectronic performance.

To further investigate the relationship between optoelectronic properties and morphology, and to verify whether the small particles induce crystallization of GPF, we next examine the films using grazing-incidence wide-angle X-ray scattering (GIWAXS). The 2D GIWAXS pattern for pristine GPF is presented in Supplementary Fig. 24, and the 1D q_z_ profiles for pristine GPF and the blended films are shown in Supplementary Fig. 25 A significant shift in the π-π stacking peak was observed for the GPF: 10%SBS blend, moving from 1.4 Å⁻¹ (corresponding to a π-π stacking distance of 4.49 Å) to 1.52 Å⁻¹ (corresponding to 4.13 Å). This shift suggests enhanced crystallinity and stronger π-π stacking interactions. These structural enhancements help explain the improved optoelectronic performance of the SBS-doped blended films. A schematic illustration of the proposed mechanism is presented in Fig. 3b. Pristine GPF films, due to their tightly conjugated structure, are prone to cracking under tensile stress. This rigid structure limits molecular stretchability, facilitating fracture formation. Incorporating SBS leads to the formation of a beneficial elastic microphase structure. This engineered microphase separation enhances the crystallinity of GPF, potentially by promoting stronger π-π stacking within the GPF phase itself, thus improving the optoelectronic performance. Furthermore, the continuous elastic SBS microphase acts as a stress dissipation center during tensile deformation. By redistributing stress throughout the blend, elastic SBS microphase alleviates the tensile stress on conjugated GPF, significantly enhancing the stretchability of the blended films. Through the optimized elastic microphase structure, substantial improvements in both the optoelectronic property and stretchability of the blended films are simultaneously achieved. Due to their three-dimensional phase separation, SIS and SEBS tend to accumulate more at the top or bottom surfaces. During stretching, cracks can initiate preferentially at one of these GPF-rich interfaces. These initial cracks then propagate throughout the entire three-dimensional film, ultimately leading to film fracture. This failure mechanism is consistent with our earlier FOW and FOE test results.

To further validate the universality of our elastomer selection method, we introduced three elastomers—SBS, SIS, and SEBS—into another polymer emissive material, Super Yellow (SY). The surface energy of SY was measured to be 36.73 mN m^–1^ (Supplementary Fig. 26 and Supplementary Table 1). This value closely matches SEBS, correlating with the smallest χ_SY-SEBS_ parameter. Among all the investigated elastomers, the SY blend with SEBS achieved the best-performing OLED devices, demonstrating considerable improvements in optoelectronic and mechanical characteristics (Supplementary Figs. 27–29 and Supplementary Table 4). Conversely, devices based on blended films with SIS and SBS exhibited notable performance drops. This confirms the applicability of our proposed approach for selecting elastomers based on miscibility.

### Stretchable transparent electrode

High-performance STEs crucial for *is*-OLEDs. AgNWs are a promising candidate for STEs due to their transparency and stretchability. However, AgNWs have limitations, such as poor adhesion and high surface roughness, which can impede the efficiency of electroluminescence. In our previous study, we introduced an STE composed of AgNWs embedded in thermoplastic polyurethane (TPU), designated as AT STE.^55^ Despite its potential, this approach still faces challenges. Issues such as significant conductivity loss from fractures and incomplete material transfer during peeling-off persist. Additionally, the surface roughness of the AT electrode remains insufficient to meet the requirements of thin-film OLED devices. The AT STE still necessitates UVO treatment before subsequent spin-coating, which can have adverse effects on the electrode’s performance.

**Fig. 4:**
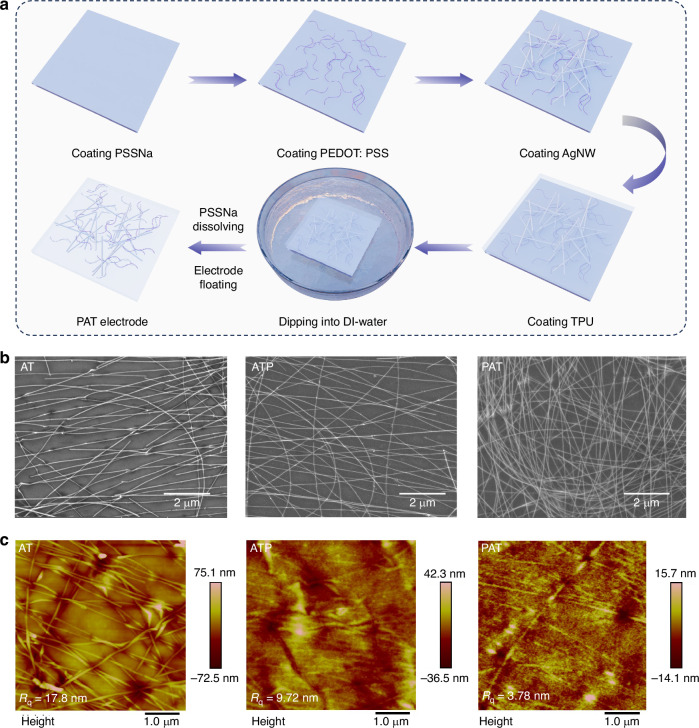
Fabrication process and morphological characterization of stretchable electrodes. **a** A schematic illustration of the fabrication process for the PAT electrode. **b** SEM images of the AT, ATP, and PAT electrodes, respectively. **c** AFM height images of the ATP, AT, and PAT electrodes, respectively

To address these limitations, a novel dual-embedded STE denoted as PEDOT: PSS@AgNW@TPU (PAT), was developed. Both the PH1000 layer and the AgNW network are embedded into the TPU matrix. The fabrication process, illustrated in Fig. 4a, Supplementary Figs. 30–31 initiates the spin-coating of a water-soluble PSSNa layer onto a rigid substrate. Subsequently, a conductive PEDOT: PSS thin layer is coated onto the PSSNa layer, followed by the spin-coating of AgNWs onto the PEDOT: PSS surface. Finally, a TPU layer is spin-coated onto the AgNWs, filling the voids within the network. Upon immersion in deionized water for five minutes, the electrode detaches and floats to the surface of the water as the PSSNa layer dissolves completely. This method eliminates the need for physical peeling, which can potentially damage the AgNW network and prevent a complete transfer.

The morphology of PAT, AT, and ATP electrodes (AT electrode with a PEDOT: PSS layer spin-coated after transfer) was assessed by analyzing SEM images and AFM images. As evidenced by SEM images (Fig. 4b), PAT and ATP electrodes present notably smoother surfaces than the AT electrode. Besides, in contrast to PAT electrodes, the AgNW network in both AT and ATP electrodes exhibit noticeable discontinuities and breaks after the peeling-off transfer process. This suggests that the PH1000 pre-coating in PAT provides some protection to the AgNW network during transfer. The PAT electrode displays a more complete AgNW network, with more AgNWs visible across the surface compared to the AT and ATP electrodes. The relative resistance (R/R₀) before and after peeling was found to be 1.5 for AT, and less than 1.3 for PAT, which further supports this observation (Supplementary Fig. 32). AFM analysis (Fig. 4c) provides quantitative data on surface roughness. The PAT electrode exhibits a superior smoothness with a Rq of 3.78 nm, compared to the ATP electrode (Rq = 9.72 nm) and the AT electrode (Rq = 17.8 nm). The reduced roughness of the PAT electrode minimizes charge transport barriers, facilitating more efficient lateral charge diffusion and improving the film quality of the following deposited emissive layer.

**Fig. 5:**
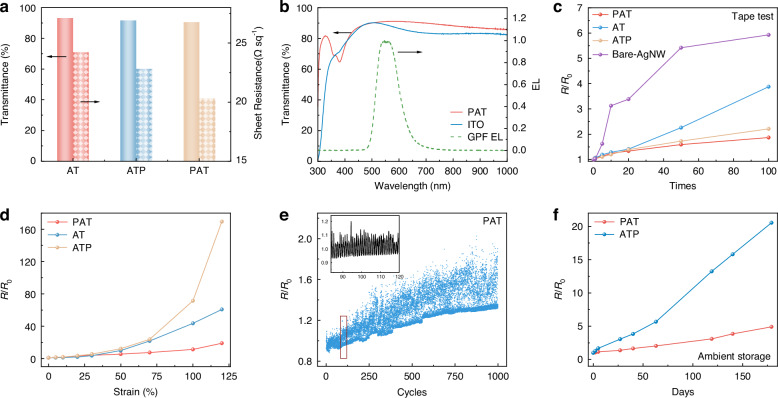
Optoelectronic and mechanical properties of stretchable electrodes. **a** Comparison of average visible light transmittance and sheet resistance for the three electrodes (PAT, AT, ATP). **b** Transmittance spectrum of the PAT electrode and ITO electrode overlaid with the EL emission spectrum of GPF. **c** Tape test results compare three stretchable electrodes (PAT, AT, ATP) and an AgNW electrode coated on a glass substrate. The data was collected from 3 independent devices. **d** Resistance variation of the three electrodes (PAT, AT, ATP) under different tensile strain. The data was collected from 3 independent devices. **e** Long-term mechanical stability of PAT electrode under continuously stretching-releasing cycles with maximum tensile strain of 20%. **f** Stability of the PAT and ATP electrodes under ambient storage conditions. The data of PAT and ATP was collected from 4 independent devices, respectively

Following the establishment of the distinct morphological characteristics of the three electrodes, their optoelectronic and mechanical performance was then examined. First, the optical transmittance and electrical resistance of the three STEs were measured (Fig. 5a). All three STEs exhibited similar average visible light transmittance, reaching approximately 90%. However, the resistance of PAT was significantly lower compared to AT and ATP, measuring 83% and 89% of their respective resistances. The transmittance of PAT STE was also compared to a commercially available rigid ITO electrode and the electroluminescence (EL) spectrum of a green OLED (Fig. 5b), demonstrating similar transmittance to ITO and minimal interference with light emission with an average transmittance of 90.2% in the emission region (480-650 nm).

The adhesion of the electrode was evaluated using a tape test (Fig. 5c), with the PAT electrode showing a significant improvement in adhesion compared to the AT and ATP electrodes. The PAT electrode demonstrated a minimal resistance change of 1.86 (ratio of the resistance at stretch to the initial) over 100 cycles. In comparison, the resistance change was higher for AT (3.88), ATP (2.21), and bare AgNWs (5.93). This suggests strong adherence, which is essential for ensuring better mechanical stability during operation and transfer. Figure 5d presents the relative resistance change of the PAT, AT, and ATP electrodes as a function of tensile strain. The PAT electrode exhibits a minimal increase in resistance at lower strain levels. It remains relatively stable up to 100% strain. At 120% strain, the resistance increases to approximately 18 times its original value. In contrast, at 120% strain, the resistance of the AT and ATP electrodes increased by factors of approximately 60 and 170 times their original resistances, respectively.

The dynamic stretching performance of the PAT electrode was evaluated (Fig. 5e) by monitoring its real-time resistance change during 1000 cycles of stretch-release with a maximum strain of 20%. The PAT electrode showed only a slight resistance increase, reaching 1.35 times its initial resistance after 1000 cycles (Supplementary Fig. 33). In contrast, the AT and ATP electrodes reached 2.52 and 2.15 times their initial resistances, respectively. The long-term ambient stability of the electrodes was investigated (Fig. 5f), where the PAT electrode demonstrated minimal resistance increase over prolonged exposure. After 180 days of ambient exposure, the resistance of the PAT electrode increased by approximately 5 times its original value, compared to the ATP electrode, which increased by approximately 21 times its original value. This is primarily attributed to the superior encapsulation effect achieved by the complete embedding of AgNWs within the TPU matrix, as well as to the enhanced interfacial adhesion with PEDOT: PSS.

The novel dual-embedded PAT electrode was fabricated using a dual-embedded structure. It has improved surface morphology, enhanced electrical performance, improved adhesion, superior mechanical stability, and enhanced long-term ambient stability. A more complete AgNW network and lower surface roughness, resulting from the combined effect of the unique dual-embedded structure, pre-buried PH1000, and a water-soluble PSSNa layer, eliminates the need for direct physical peel-off. These make it an excellent candidate for next-generation wearable electronics devices.

### *Is*-OLED

**Fig. 6:**
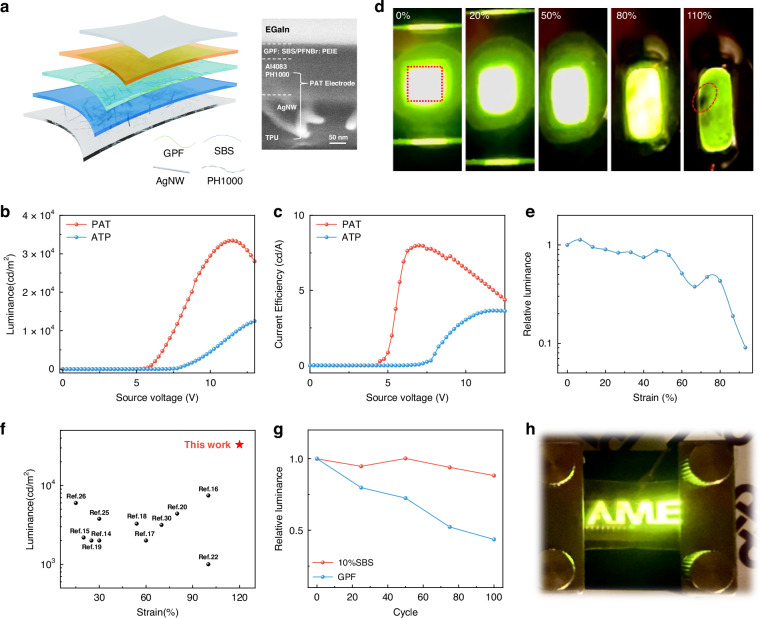
Structural, optoelectronic and mechanical performance characterization of the intrinsically stretchable OLED. **a** A schematic illustration of the *is*-OLED structure and cross-section SEM picture. **b** Luminance and **c** current efficiency versus voltage curves for *is*-OLEDs using two stretchable electrodes (ATP, PAT). **d** An image of the device under varying strain from 0% to 110%. **e** Variations in the *is*-OLED’s relative luminance at 9 V under different strain levels. **f** Comparison of luminance and strain capabilities of the *is*-OLEDs reported here versus previous studies. **g** Relative luminance changes under 100 repeated stretching-releasing cycles (15% strain) for the *is*-OLED based on a GPF: 10%SBS blended film, compared to pristine GPF. **h** An image of the *is*-OLED operating under 30% strain

Consequently, the *is*-OLED was fabricated using a structure of PAT/PEDOT: PSS (4083)/GPF: 10%SBS/PFNBr: PEIE/EGaIn. (Fig. 6a, Supplementary Figs. 34–35). Spray-coated eutectic gallium-indium (EGaIn) was employed as the stretchable top electrode which is a promising alternative to evaporated metal electrodes.^56,57^ The device based on EGaIn, and Ag top electrode exhibited comparable performance (Supplementary Fig. 36 and Supplementary Table 5). Figure 6b, c, and Supplementary Fig. 37 present the optoelectronic performance including luminance, current efficiency, and EQE of *is*-OLED with PAT and ATP STEs. The PAT-based *is*-OLED attained a peak luminance of 33,443 cd m^−2^, comparable to rigid OLED (85% of rigid blend OLED). The PAT-based *is*-OLED exhibited a maximum current efficiency of 7.99 cd A^-1^ and an EQE of 2.28% (both 106% of rigid blend OLED), which were twice as high as those of the ATP-based *is*-OLED. The *is*-OLED devices demonstrate exceptional performance under mechanical deformation. Figure 6d illustrates the *is*-OLED’s performance under varying strain levels, from 0% to 110%, maintaining uniform brightness up to 80% strain. Even at 110% strain, the device continues to operate with minimal loss of homogeneity, highlighting its mechanical durability and ability to withstand high deformation. The device can endure a maximum strain of 120% (Supplementary Video 1), a significant improvement over the pristine GPF device, which can only endure 20% (Supplementary Video 2). The device failure at 120% strain is attributed to the combined effects of the significant resistance increase in the PAT electrode and the severance of charge transport pathways within the emissive layer.

Figure 6e shows the real-time relative luminance change of the *is*-OLED at 9 V during stretching, where the device maintains over 50% of its initial luminance at 60% strain. This highlights the *is*-OLED’s capability to operate effectively even under significant mechanical deformation. The operational stability of the stretchable OLED under mechanical deformation was evaluated by monitoring its relative luminance during a stretching-releasing cycle up to 40% strain (Supplementary Fig. 38). Compared to previous intrinsically stretchable LED devices, our *is*-OLED simultaneously achieves high luminance and excellent stretchability (Fig. 6f and Supplementary Table 6). Moreover, the durability of the *is*-OLED is further demonstrated in Fig. 6g and Supplementary Fig. 39, where the device with the GPF: SBS blended emissive layer retains 88% of its initial luminance after 100 cycles of dynamic stretching at 15% strain (Fig. 6g and Supplementary Video 3). However, under the same conditions, the OLED using a pristine GPF emissive layer retains only 43% of its initial luminance, highlighting the improved mechanical stability conferred by SBS doping. To further assess robustness under extreme deformation, we conducted cyclic testing at 40% strain in an ambient atmosphere. The device retained 67.65% of its initial luminance after 100 cycles (Supplementary Fig. 40 and Supplementary Table 7). The *is*-OLED developed in this study exhibits both record luminescence and ultimate elongation demonstrating its superior potential for wearable applications. The image in Fig. 6h depicts the *is*-OLED patterned with the words “AME” operating effectively at 30% strain, clearly demonstrating its potential for practical applications by maintaining stable operation and high optical performance even under deformation.

## Discussion

This study developed a systematic strategy for developing high-performing *is*-OLED. On one hand, through an elastic-microphase-engineering approach incorporating SBS-derivative elastomers, the emissive layer with simultaneously improved optoelectronic and mechanical properties was achieved. The miscibility between emissive materials and elastomers is elucidated to be the decisive factor which regulates the three-dimensional microphase separation in both lateral and vertical directions for simultaneously achieving high photoelectronic and mechanical performance. On the other hand, we designed dual-embedded PAT STE with optimized conductivity, surface roughness, stretchability, and stability. As a result, *is*-OLED based on GPF: 10%SBS blended film and PAT STE demonstrates record luminance (33433 cd m^–2^), excellent maximum tensile strain (120%), and high long-term mechanical stability by maintaining approximately 90% of its initial luminance after 100 stretching and releasing cycles at 15% strain. Our work provides novel insights and approaches for material selection, and performance optimization in stretchable OLEDs. We also believe that this elastic-microphase-engineering strategy and stretchable electrode technology are applicable to other thin-film LED systems, enabling the development of high-performance stretchable and wearable displays.

## Materials and methods

### Materials

TPU Elastollan 685 A was purchased from Basf. The silver nanowires (AgNWs) dispersion in isopropyl alcohol (IPA) (D:25-35 nm, L: 30–60 μm, 1 wt%) is purchased from Zhejiang Kechuang Advanced Materials Technology. Poly(3,4-ethylenedioxythiophene): poly(styrenesulfonate) (PEDOT: PSS PH1000) was purchased from Xi’an Baolaite Optoelectronic Devices Co., Ltd. Poly(3,4-ethylenedioxythiophene) : poly(styrenesulfonate) (PEDOT: PSS AI4083) was purchased from Heraeus Deutschland GmbH & Co. KG. The GPF is green poly(9,9-dialkylfluorene) derivative (PF Green B, The Dow Chemical Company, Lot MM010919) devices, a toluene solution of PF Green B with a concentration, which was utilized following the material specifications described in our reference paper: “Visualizing the ‘Hidden’ Triplet–Triplet Fusion Process to Fluorescence in Typical Organic/Polymer Light-Emitting Diodes” (Supplementary Fig. 41).^50^ Toluene, methanol, DMF, PFNBr, polyethyleneimine ethoxylated (PEIE), PSSNa, styrene-butadiene-styrene (SBS), styrene-isoprene-styrene (SIS), and styrene-ethylene-butylene-styrene (SEBS) were purchased by Sigma-Aldrich.

### Experimental details

#### PAT electrode fabrication

The fabrication of the PAT electrode began with the spin-coating of a 10 mg/mL PSSNa solution onto pre-cleaned glass substrates at 5000 rpm, followed by annealing at 150 °C for 5 min. Subsequently, a layer of conductive PEDOT: PSS (PH1000) was spin-coated at 2000 rpm and annealed at 150 °C for 10 min to bridge the AgNWs for lateral transport and planarize the electrode surface. A 1:1 isopropyl alcohol diluted silver nanowire solution was then spin-coated at 3000 rpm and annealed at 150 °C for 10 min. Next, a 300 mg/mL solution of TPU in DMF was spin-coated at 1000 rpm and annealed at 60 °C for 4 h. The resulting multi-layered structure was then immersed in deionized water for 10 min to facilitate the release of the electrode, after which the floating PAT electrode was transferred onto a fresh glass substrate and dried at 100 °C for 5 min.

### Fabrication of ISOLEDs

Initially, solutions of varying concentrations of SBS, SIS, and SEBS were prepared in toluene. A 10 mg/mL solution of GPF was then prepared in the respective elastomer solution. Additionally, a solution of 0.2 wt% PFNBr and PEIE at a 1:1 ratio was prepared in methanol. Subsequently, PEDOT:PSS (AI 4083) was spin-coated onto the previously fabricated PAT electrodes at 3000 rpm and annealed at 100 °C for 10 minutes to serve as the hole injection layer. The substrates were then transferred to a nitrogen-filled glovebox. Inside the glovebox, a mixture of GPF and the respective elastomer solution was spin-coated at 2500 rpm and thermally annealed at 65 °C for 10 min. This was followed by the spin-coating of the PFNBr:PEIE solution at 4000 rpm, with a subsequent annealing step at 100 °C for 5 min. The EGaIn top electrode was deposited via a spray-coating technique conducted in a fume hood under ambient atmosphere. A commercial spray gun connected to a high-pressure nitrogen gas source was utilized to atomize the liquid metal. The active area pattern was defined using laser-etched aluminum shadow masks. Silver paste was applied to the two ends of the PAT electrode to serve as contact points.

For the fabrication of devices based on silver electrodes, a 100 nm Ag film was deposited by thermal evaporation through a shadow mask. The active area of these devices was 0.04 cm². For devices fabricated on ITO-coated glass substrates, patterned ITO substrates were first ultrasonically cleaned in a detergent aqueous solution, deionized water, acetone, and isopropyl alcohol (IPA) for 40 minutes each. After this cleaning process, the substrates were treated with ultraviolet ozone for 20 minutes, followed by spin-coating of PEDOT:PSS. The subsequent steps were the same as described for the fabrication on PAT electrodes (as detailed above).

### Characterizations

The fabricated *is*-OLEDs were measured using an Enlitech LQ-100 for all optoelectronic performance assessments. Transmittance and absorption were measured by Varian Cary® 300 UV-Vis spectrophotometers (Agilent Technologies). Resistance variation is conducted by an electrochemical workstation (AUTOLAB, PGSTAT 302 N). Steady-state photoluminescence (PL) and time-resolved photoluminescence (TRPL) transient decay spectra were acquired using an FLS920 PL spectrometer from Edinburgh Instruments. GIWAXS measurements were carried out with a Xeuss 2.0 SAXS/WAXS laboratory beamline using a Cu X-ray source (8.05 keV, 1.54 Å) and Pilatus3R 300 K detector. The mechanical stability (stretching) was performed by a linear reciprocating motor. AFM morphology was measured using a BRUKER Scanning Electrochemical Microscopy System. For bottom-surface morphology, the film was spin-coated on PEDOT (AI 4083) layer and then dissolved AI 4083 in deionized water. A silicon wafer was then brought into contact with the top surface of the floating film to lift it, exposing the bottom interface for AFM scanning.

### FOW measurements

The active layer solution was spin-coated on PEDOT:PSS according to the optimal device fabrication conditions. A 3 W laser with a wavelength of 355 nm was used to carve out dog-bone film (9 × 3 mm) specimens. The whole specimen was immersed in water to dissolve the PEDOT:PSS layer and the active layer film was delaminated from the glass substrate and subsequently the film was floated on the water surface. The active layer film was held with PDMS-coated grips via van der Waals adhesion for measurements. The tensile test was performed by applying a tensile strain using a linear displacement stage at a speed of 0.5 mm/min until fracture occurred at the specimen.

## Supplementary information

SUPPLEMENTAL MATERIAL

Supplementary Video 1

Supplementary Video 2

Supplementary Video 3

## Data Availability

The data that support the findings of this study are available from the corresponding author upon reasonable request.
