# Supplementary material for: Intrinsically stretchable organic light-emitting-diode with high brightness and stretchability via elastic-microphase-engineered emitter and dual-embedded electrode
Source: Light Sci Appl. 2026 Mar 25;15:182. doi: 10.1038/s41377-026-02271-z (PMC13013940; doi:10.1038/s41377-026-02271-z)
Supplement: Supplementary file 1 — SUPPLEMENTAL MATERIAL [file 41377_2026_2271_MOESM1_ESM.docx]

Supplementary Information for

**Intrinsically Stretchable Organic Light-emitting-diode with High Brightness and Stretchability via Elastic-Microphase-Engineered Emitter and Dual-Embedded Electrode**

Zhen Lu^1,2^, Jiaming Huang^1,*^, Qiong Liang^1^, Kuan Liu^1^, Dongyang Li^1^, Jiayuan Zhu^3^, Cenqi Yan^3^, Yaokang Zhang^4^, Heng Liu^5^, Xinhui Lu^5^, Jianjun Tian^6^, Zi Jing Wong^2,*^ and Gang Li^1,7,*^

^1^Department of Electrical and Electronic Engineering, Research Institute for Smart Energy (RISE); Photonic Research Institute (PRI), The Hong Kong Polytechnic University, Hung Hom, Kowloon. Hong Kong, China.

^2^School of Electronic Science and Technology, Eastern Institute of Technology, Ningbo 315200, China.

^3^College of Polymer Science and Engineering, State Key Laboratory of Polymer Materials Engineering, Sichuan University, Chengdu 610040, China.

^4^College of Chemistry and Environmental Engineering, Shenzhen University, Shenzhen 518055, P. R. China.

^5^Department of Physics, The Chinese University of Hong Kong, Shatin, Hong Kong, China.

^6^Institute for Advanced Materials and Technology, University of Science and Technology Beijing, Beijing, 100083 China.

^7^Guangdong-Hong Kong-Macao Joint Laboratory for Photonic-Thermal-Electrical Energy Materials and Devices, The Hong Kong Polytechnic University, Hung Hom, Kowloon. Hong Kong, China.

*Correspondences: Gang Li (e-mail: [gang.w.li@polyu.edu.hk](mailto:gang.w.li@polyu.edu.hk)).

Zi Jing Wong (e-mail: zijing@eitech.edu.cn)

Jiaming Huang (e-mail: jiamhuang@polyu.edu.hk)


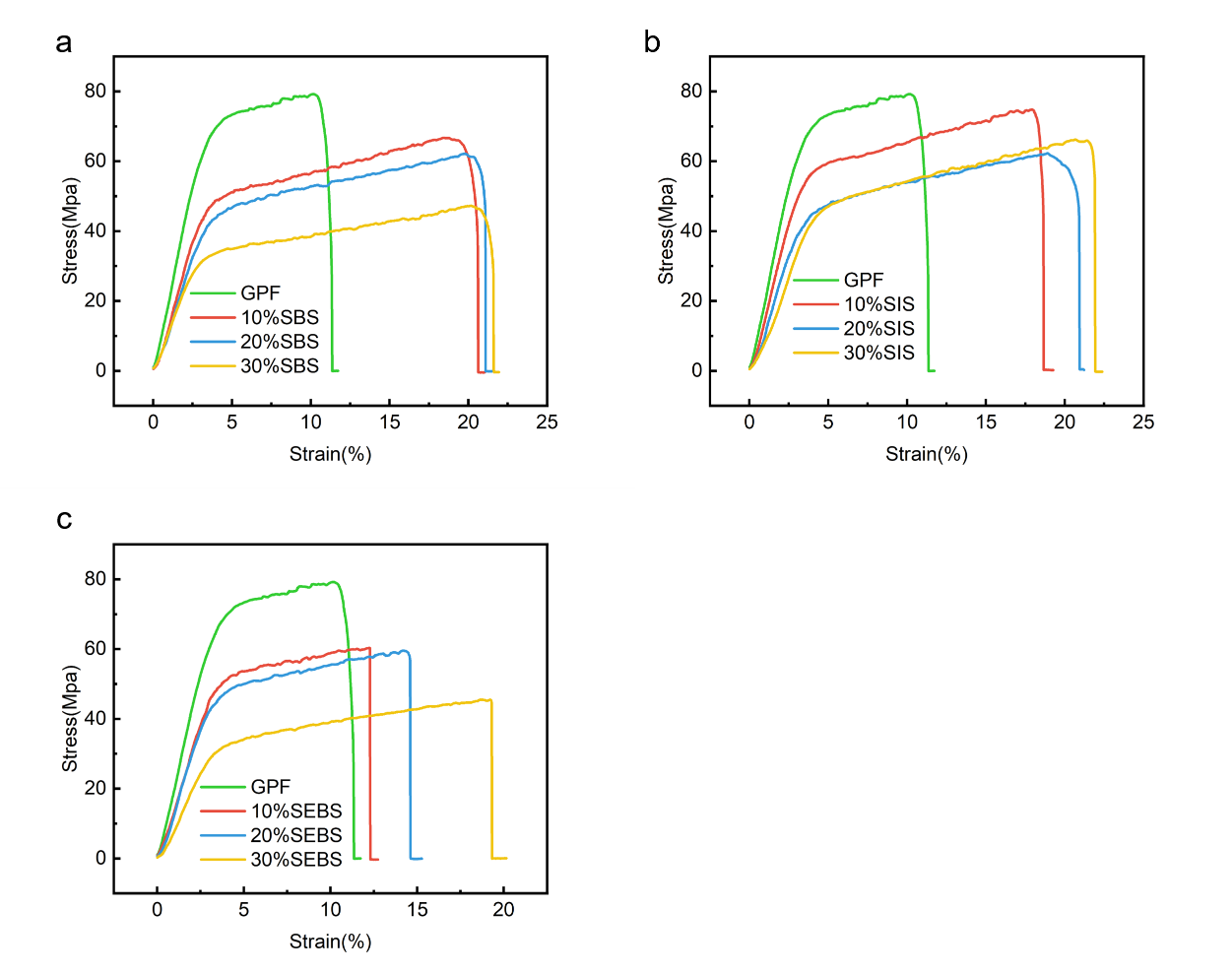
**Supplementary Fig 1.** Tensile stress curves for the film-on-water (FOW) configuration, comparing pristine GPF and GPF films doped with varying concentrations of each elastomer: (a) SBS, (b) SIS, and (c) SEBS.


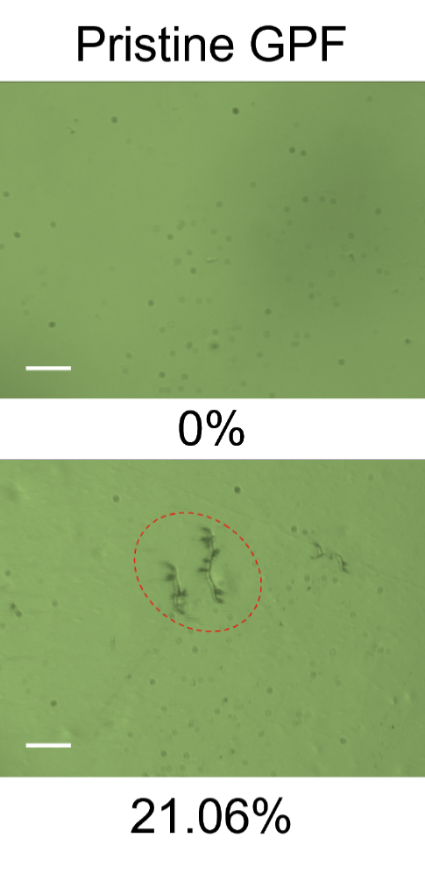


**Supplementary Fig 2.** Optical image of GPF film under strain at crack on set, the tensile strain was applied along the horizontal axis. Scale bar: 5 μm.


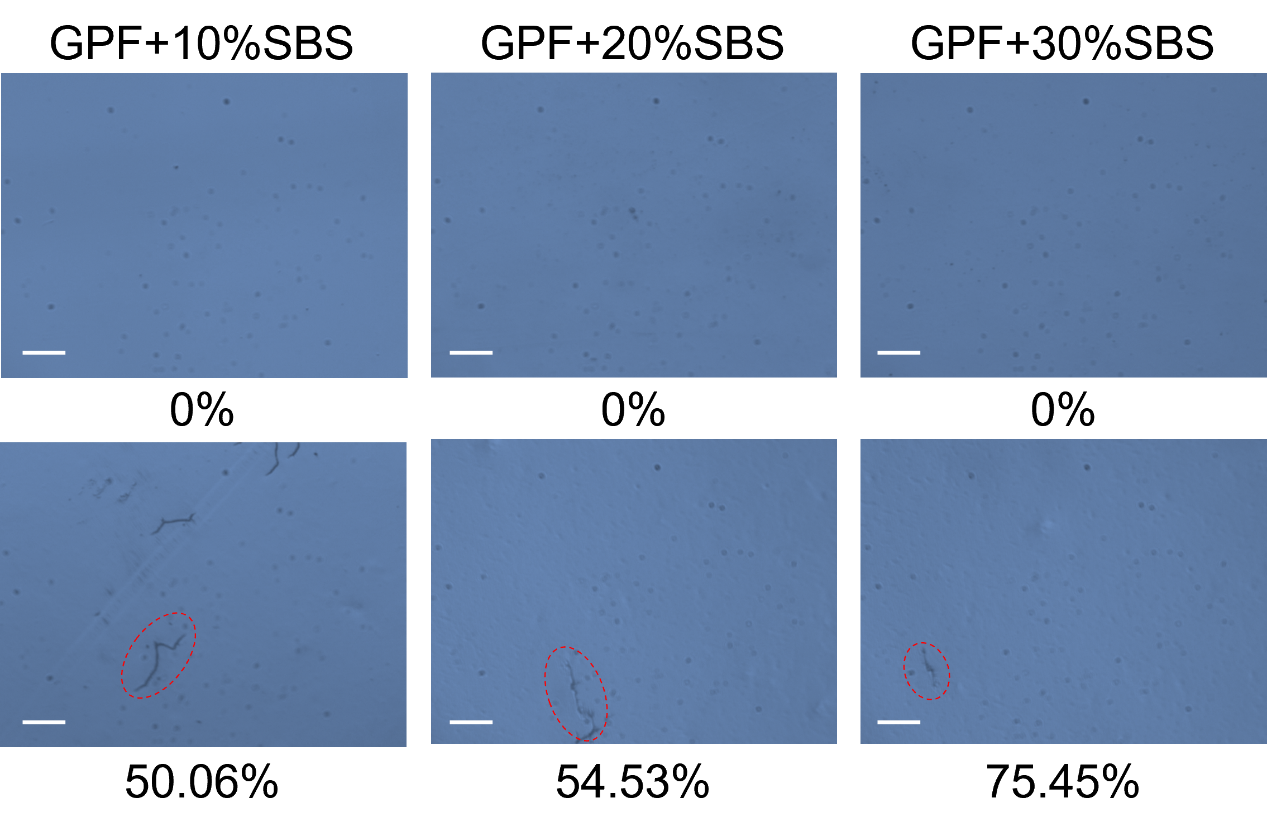


**Supplementary Fig 3.** Optical images of GPF/SBS films with increasing SBS amount under different strains at crack onset, the tensile strain was applied along the horizontal axis. Scale bar: 5 μm.


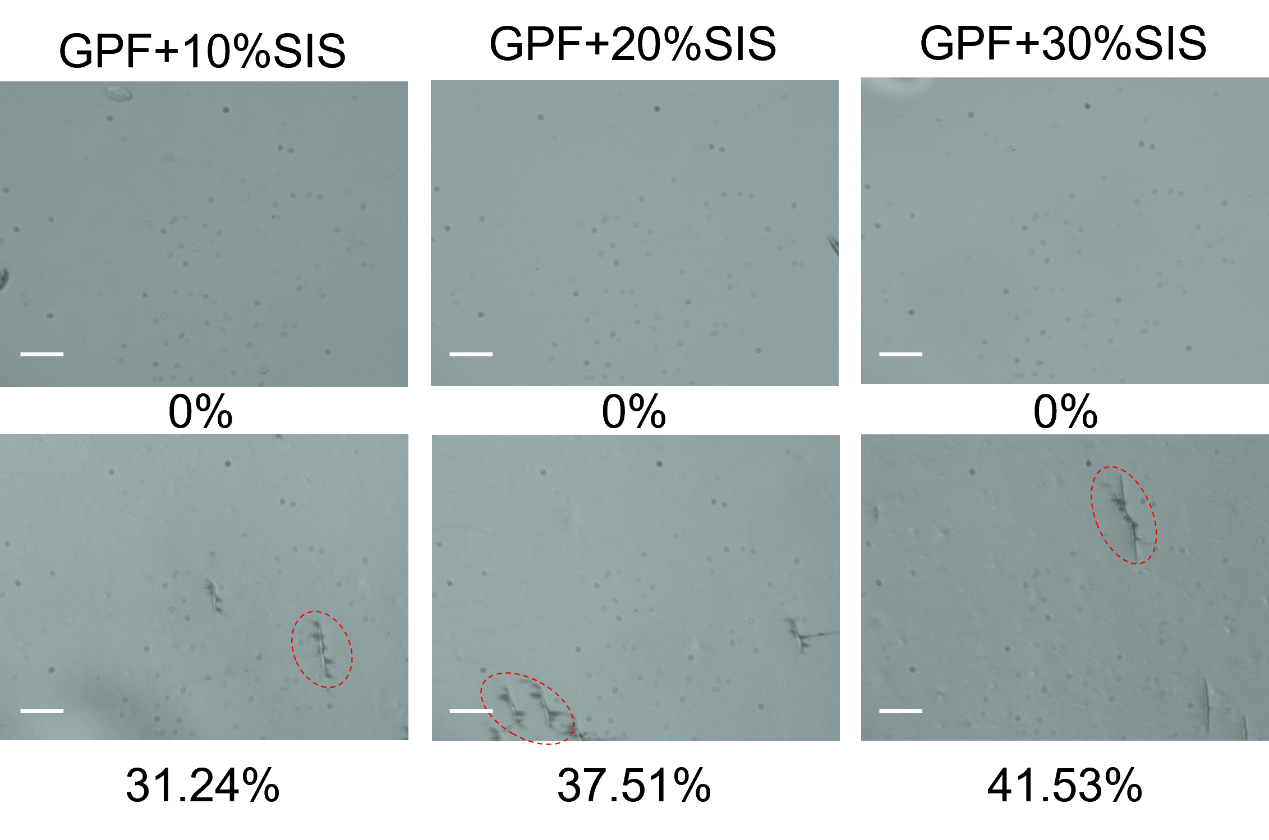


**Supplementary Fig 4.** Optical images of GPF/SIS films with increasing SIS amount under different strains at crack onset, the tensile strain was applied along the horizontal axis. Scale bar: 5 μm.


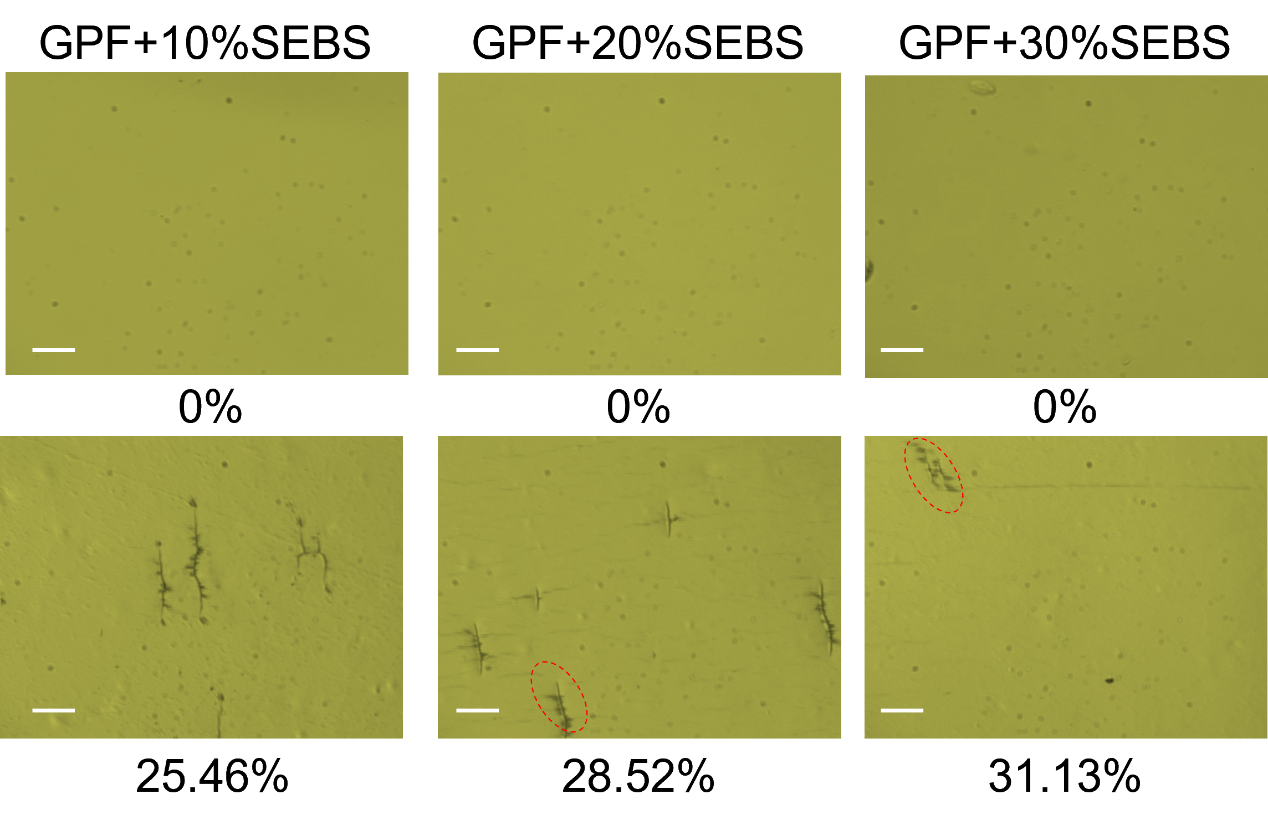


**Supplementary Fig 5.** Optical images of GPF/SEBS films with increasing SEBS amount under different strains at crack onset the tensile strain was applied along the horizontal axis. Scale bar: 5 μm.


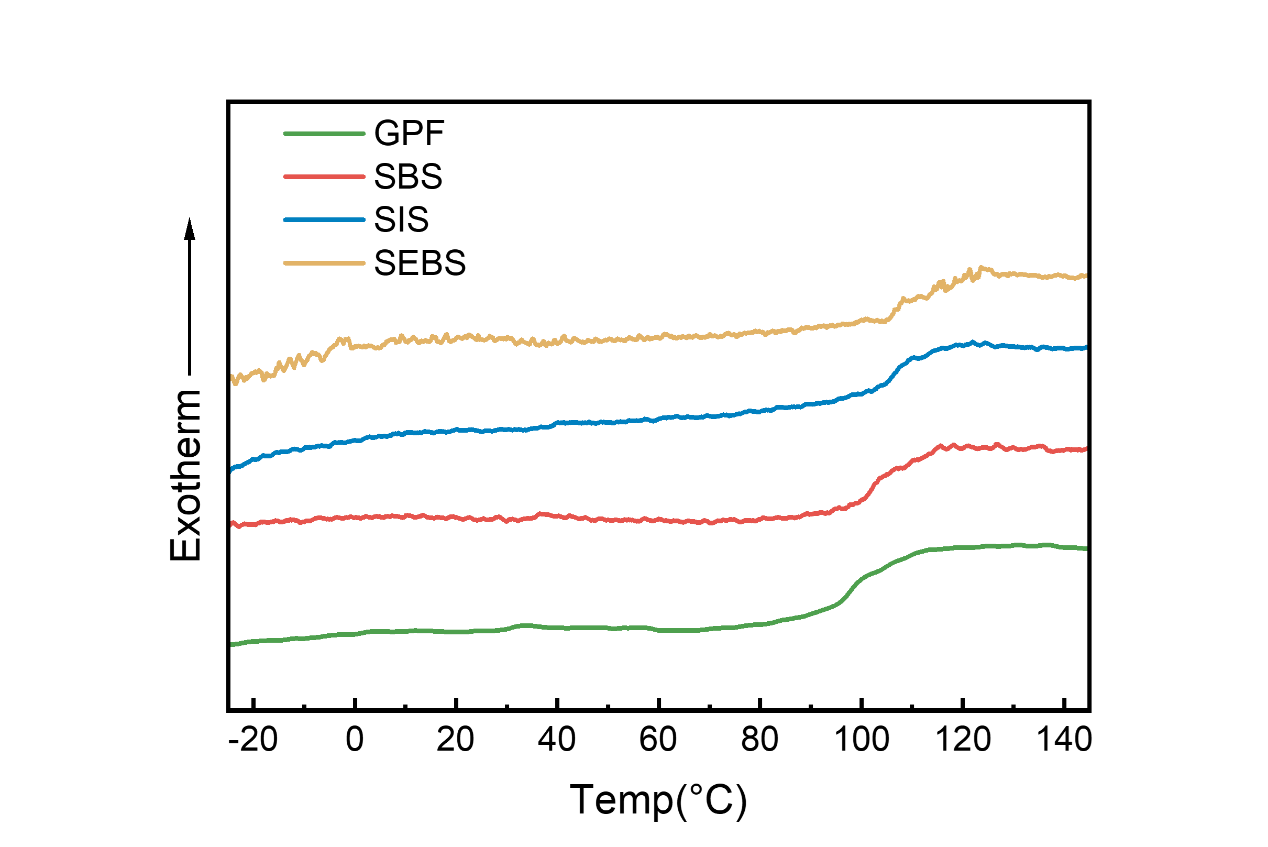


**Supplementary Fig 6.** Differential Scanning Calorimetry (DSC) thermograms of pristine GPF and GPF blended with 10% SBS, SIS, and SEBS.


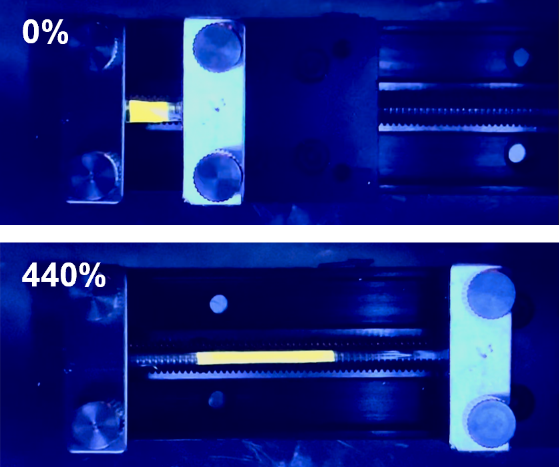


**Supplementary Fig 7.** Photoluminescence Images of 10% SBS-Doped GPF Blend Film Under Mechanical Strain from 0% to 440%.


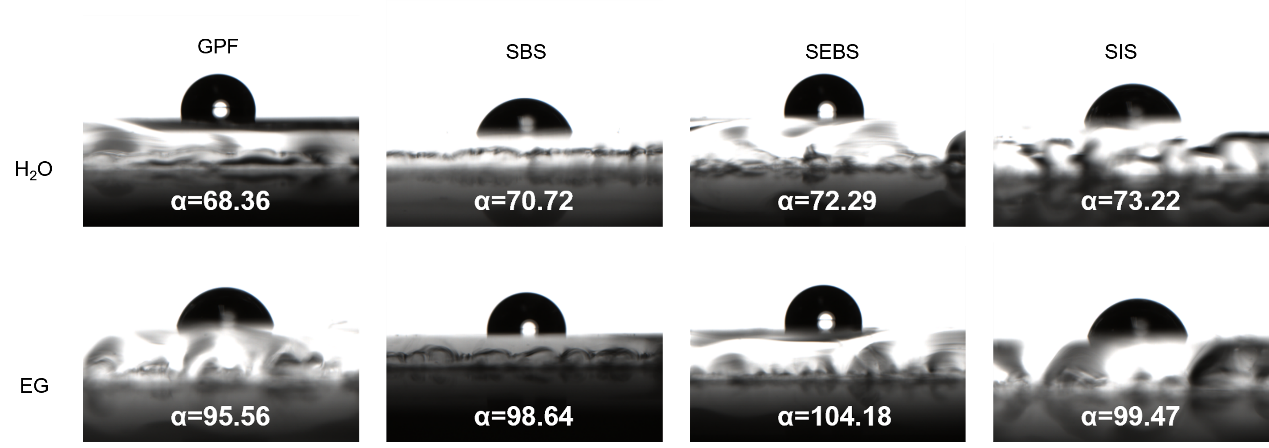


**Supplementary Fig 8.** Optical images showing the contact angles of GPF, SBS, SEBS, and SIS films, respectively.


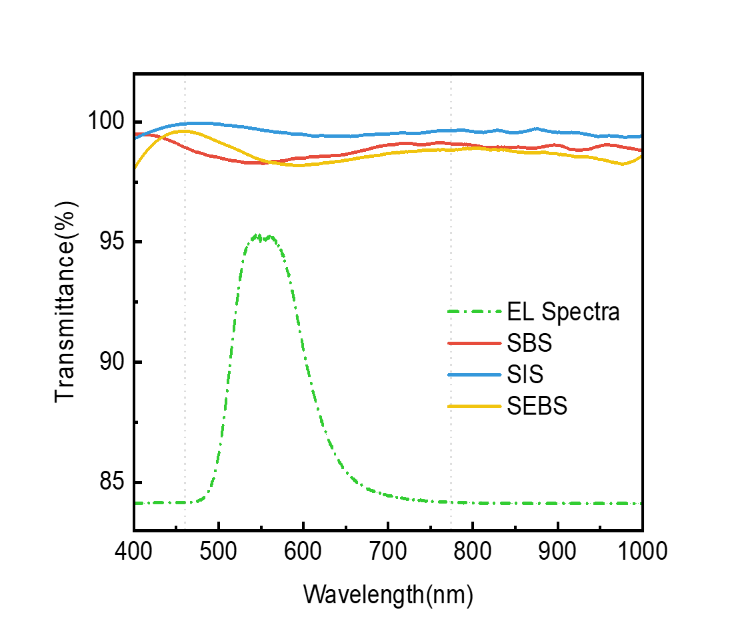


**Supplementary Fig 9.** Transmittance spectra of SBS, SIS, and SEBS in the visible wavelength range, compared with the EL spectra of the light-emitting material.


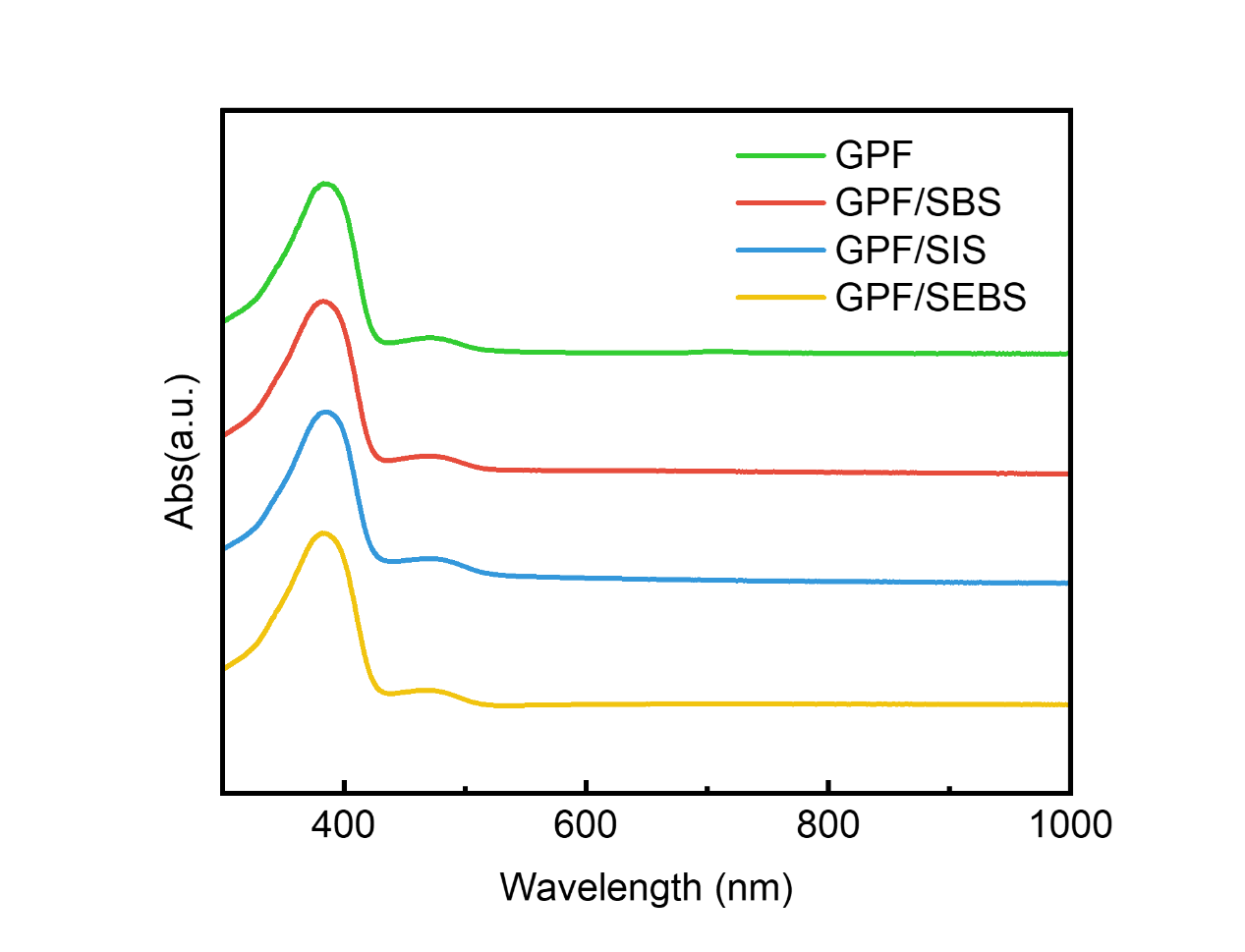


**Supplementary Fig 10.**Absorption spectra of pristine GPF and GPF doped with SBS, SIS, and SEBS.


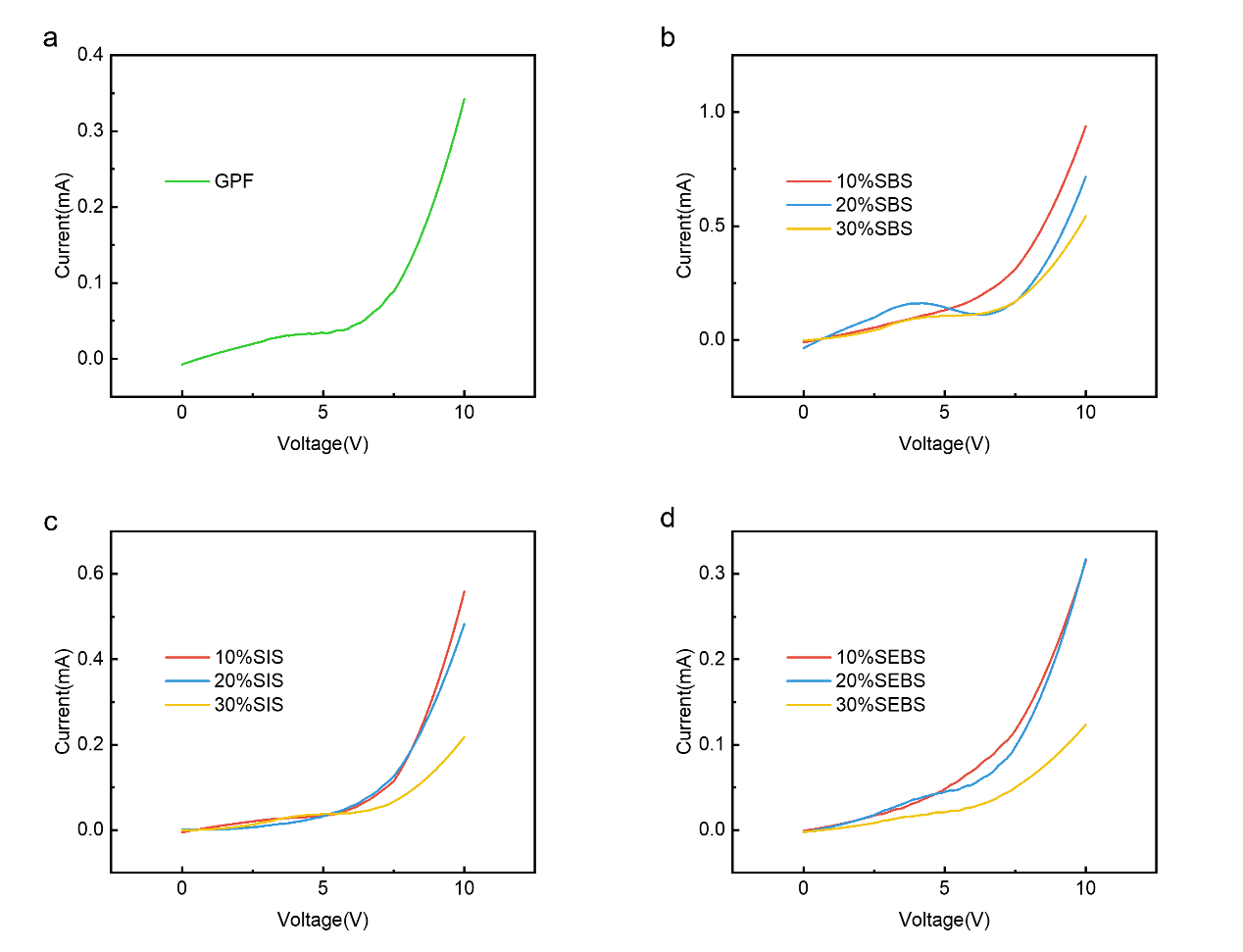


**Supplementary Fig 11.** Electron-only devices. Trap-limited electron transport in GPF/elastomer films measured in a series of ITO/ZnO/active layer/PFN-Br:PEIE/Ag electron-only devices. Active layers: (a) pristine GPF, (b) GPF:SBS, (c) GPF:SIS, and (d) GPF:SEBS.


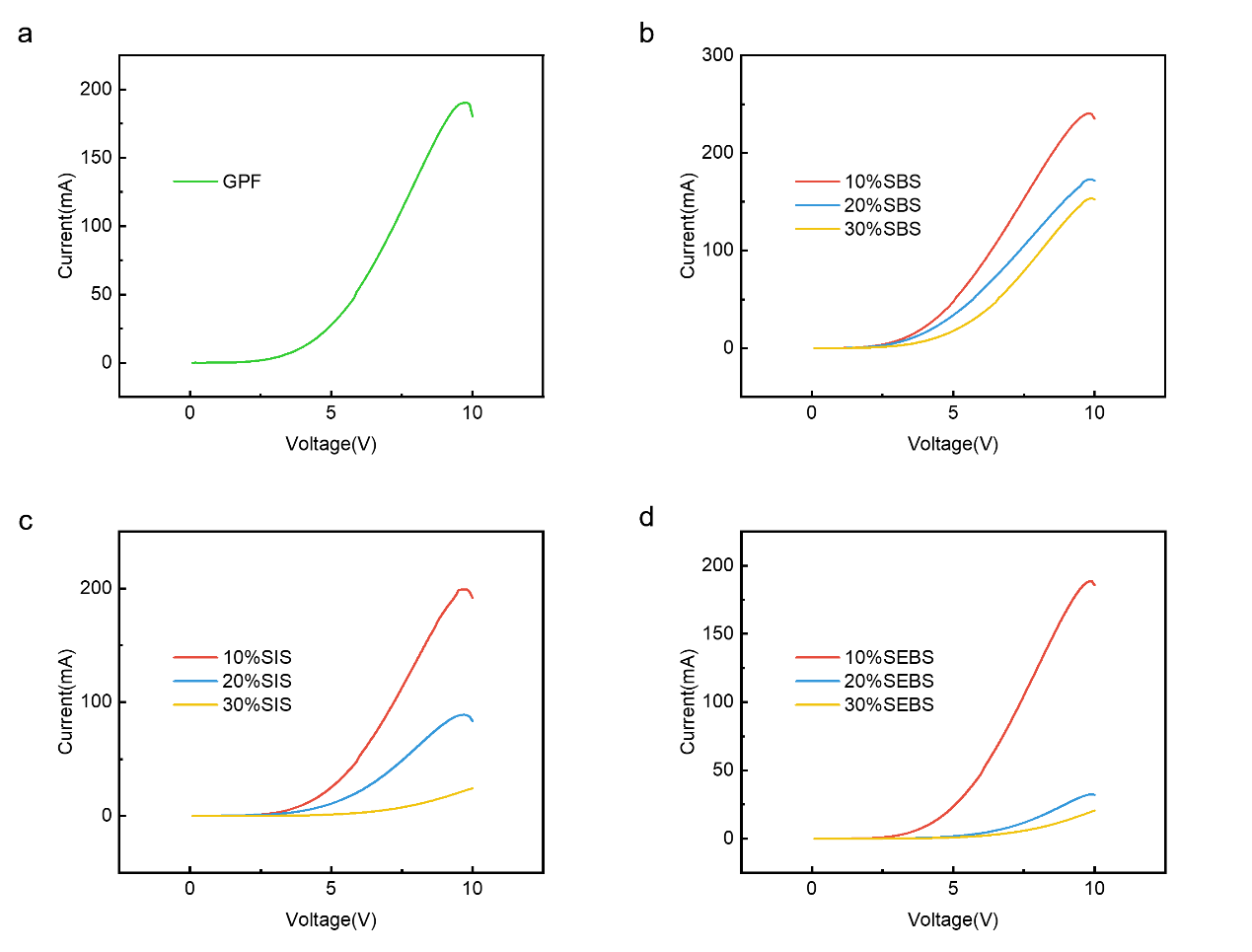


**Supplementary Fig 12.** Hole-only devices. The trap-free hole transport in GPF/elastomer films measured in a series of ITO/PEDOT:PSS/ active layer/MoO3/Ag electron-only devices. Active layers: (a) pristine GPF, (b) GPF:SBS, (c) GPF:SIS, and (d) GPF:SEBS.


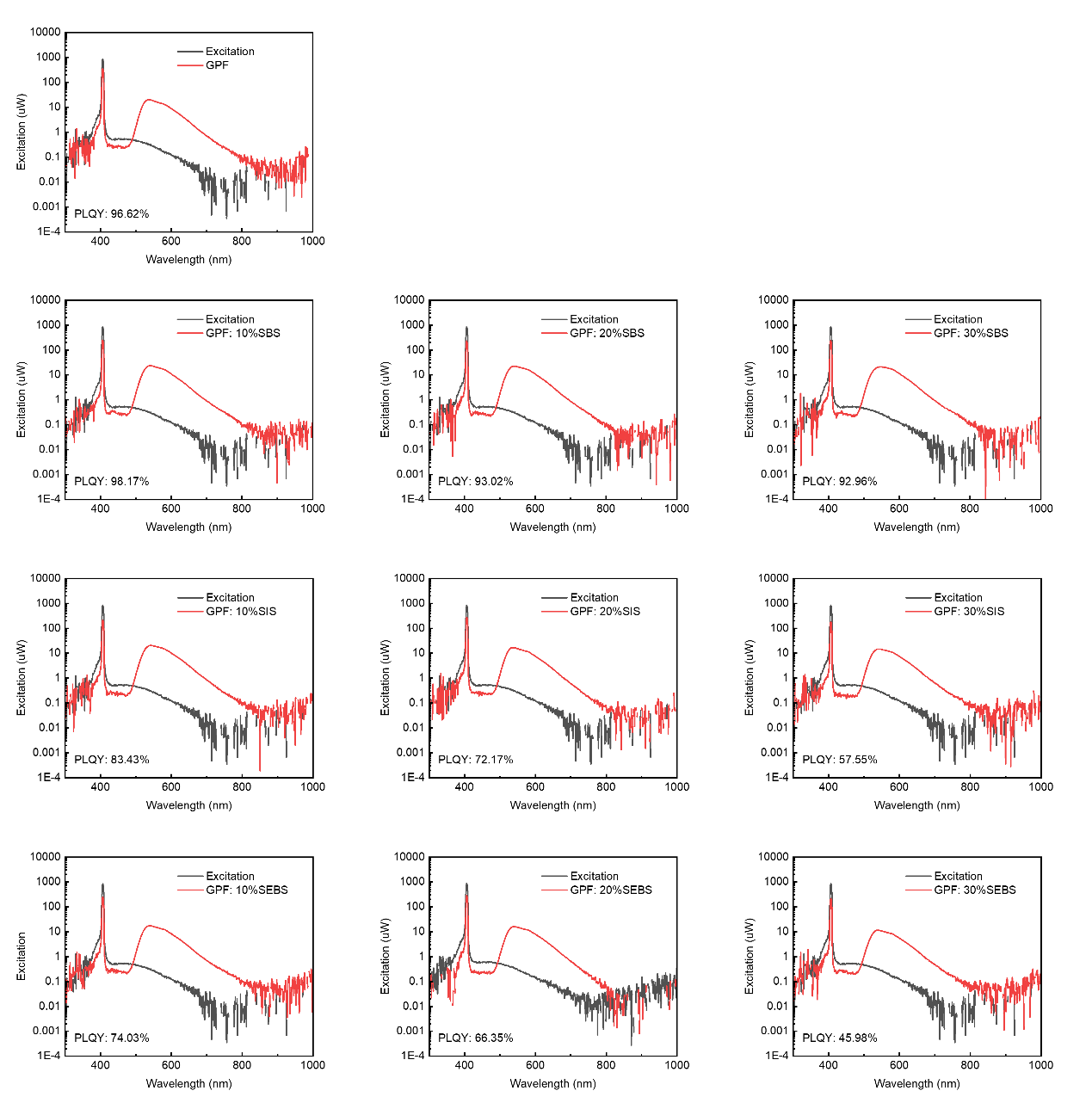


**Supplementary Fig 13**. PL spectra used for PLQY of GPF and GPF: elastomers calculation.


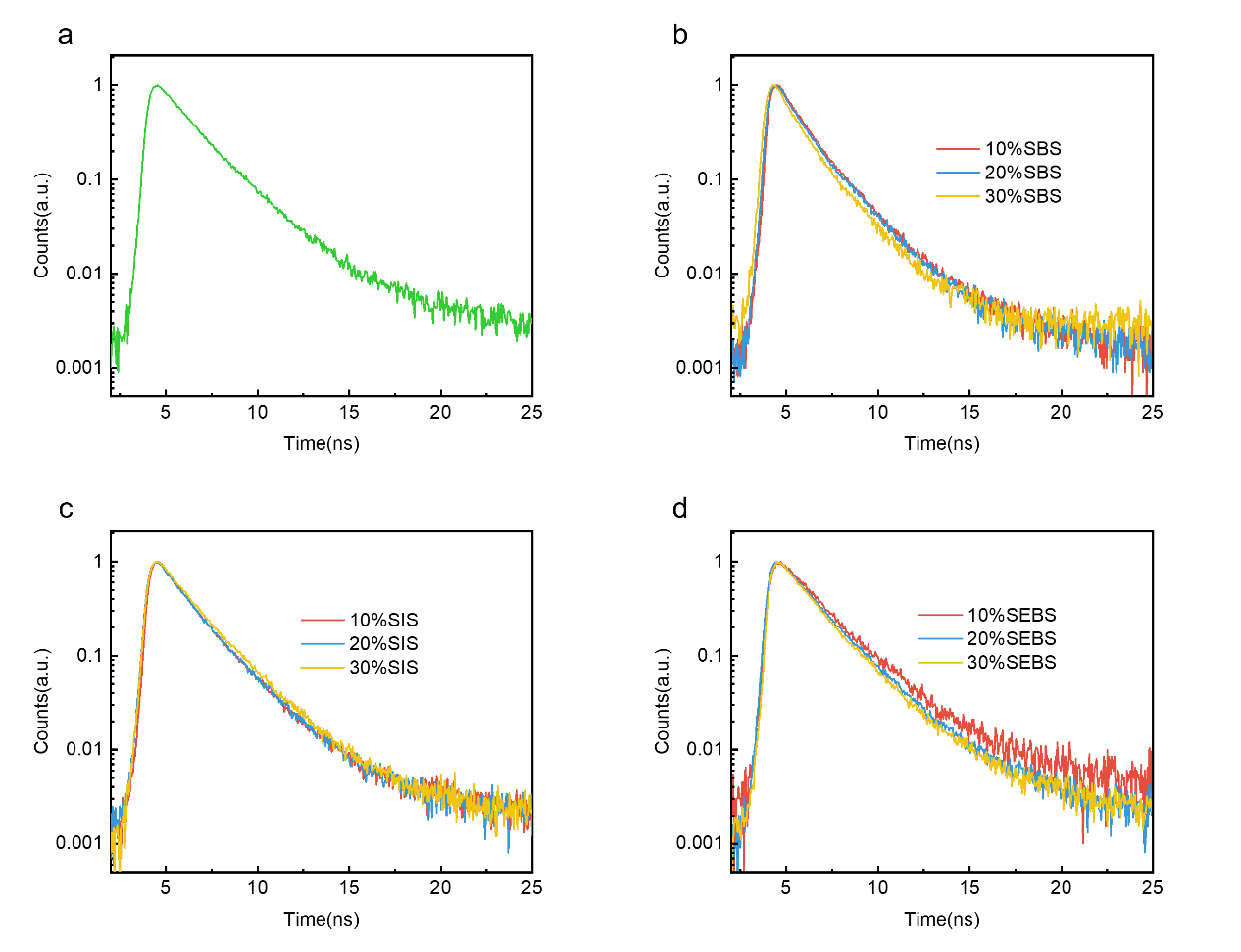
**Supplementary Fig 14.** TRPL spectra of GPF and GPF blended with varying amounts of elastomer: (a) GPF, (b) GPF:SBS, (c) GPF:SIS, and (d) GPF:SEBS.


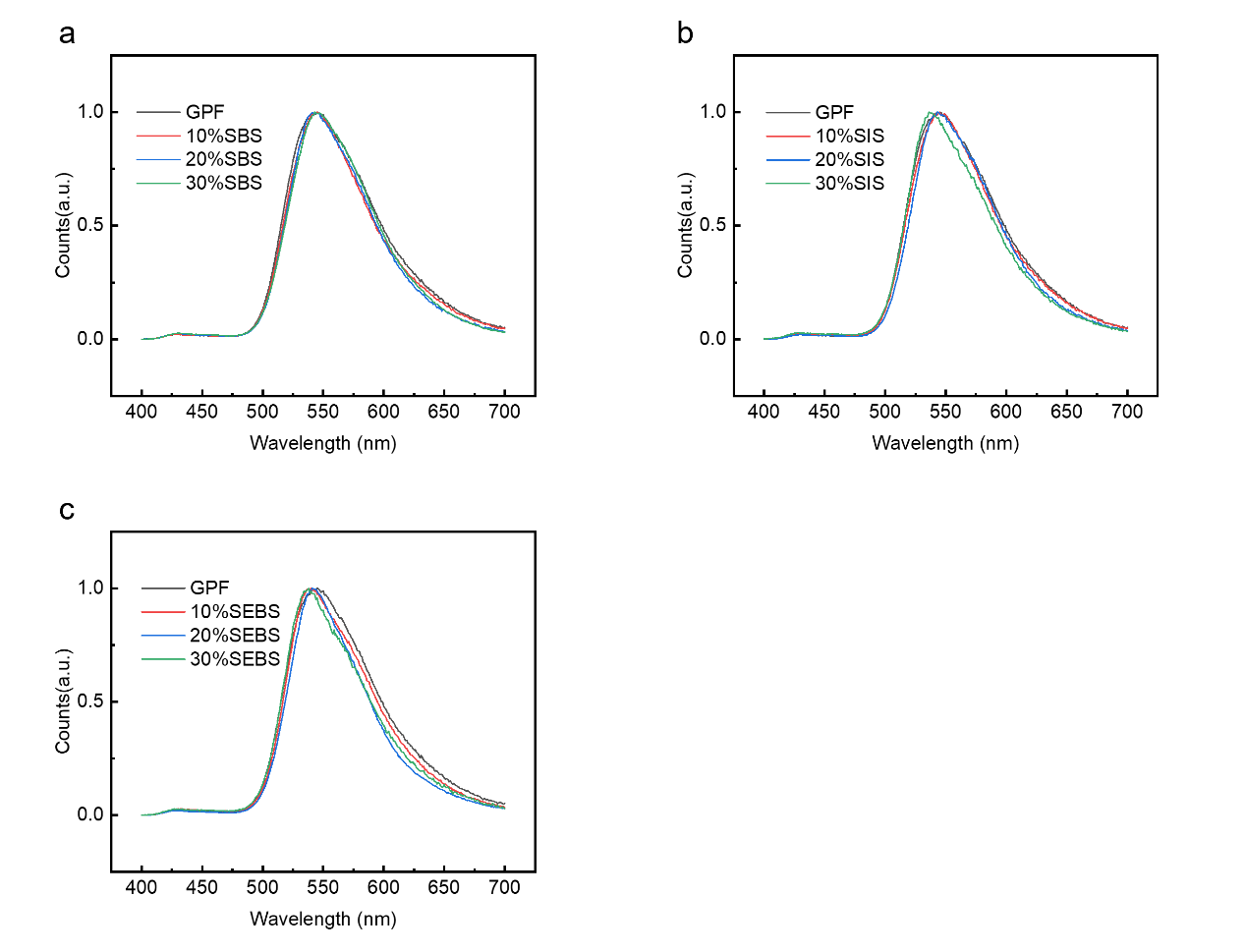


**Supplementary Fig 15.** PL spectra of GPF and GPF blended with varying amounts of elastomer: (a) GPF: SBS, (b) GPF: SIS, and (c) GPF: SEBS.


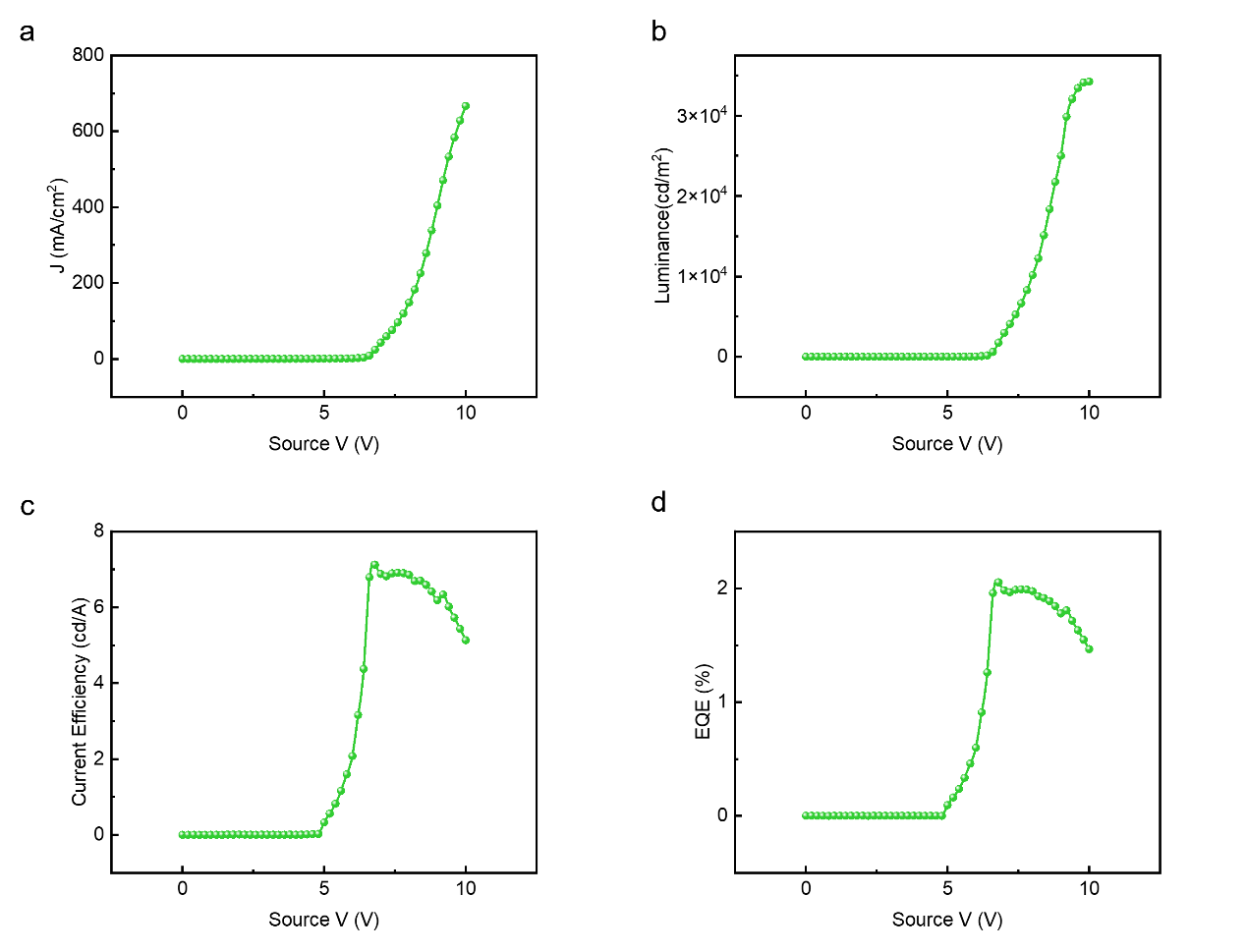


**Supplementary Fig 16.** (a) J–V curves, (b) L–V curves, (c) current efficiency curves, and (d) EQE curves for ITO/PEDOT:PSS/GPF/PFNbr/PEIE/Ag devices.


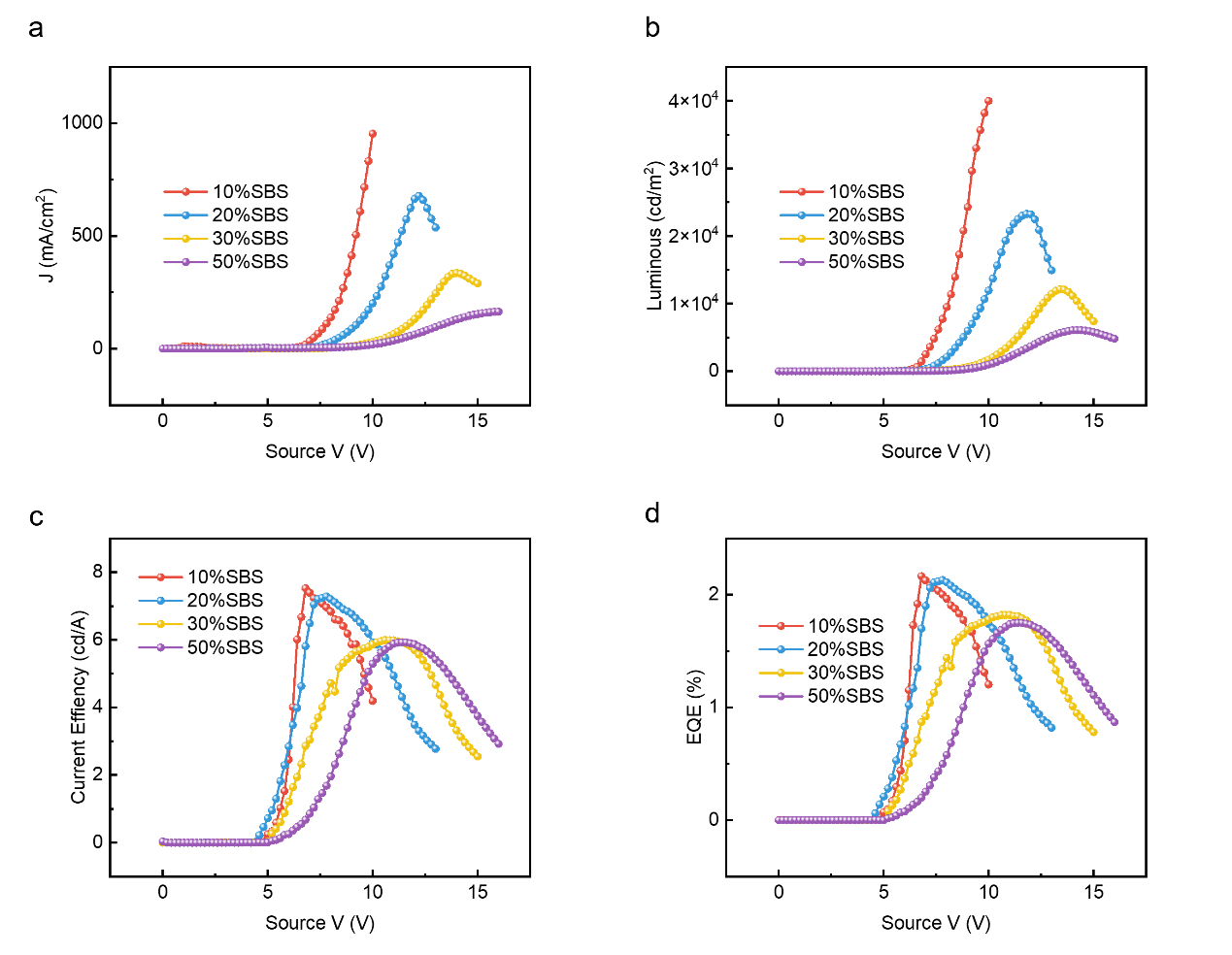


**Supplementary Fig 17.** (a) J–V curves, (b) L–V curves, (c) current efficiency curves, and (d) EQE curves of OLED devices incorporating GPF: SBS blended films with various SBS concentrations.


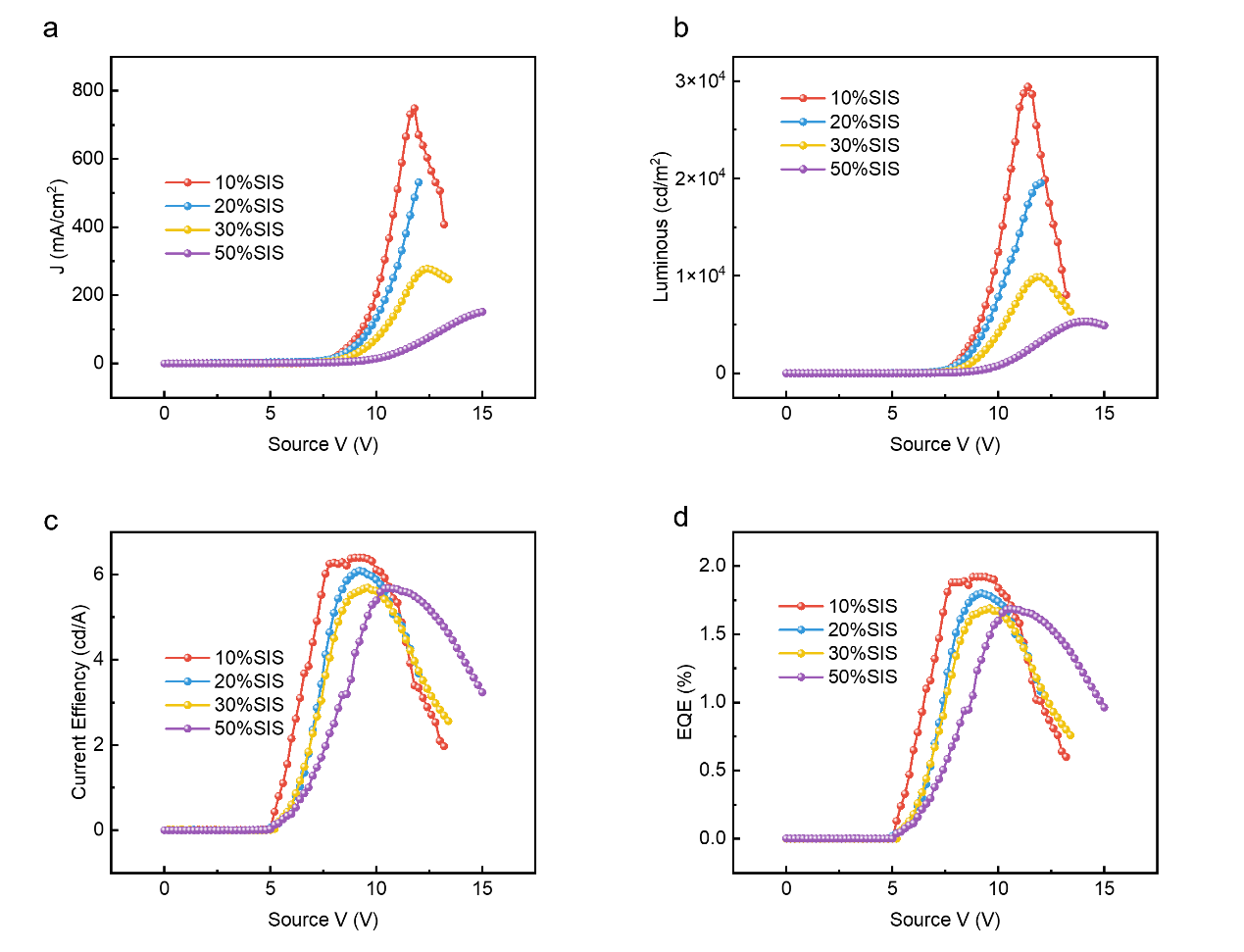


**Supplementary Fig 18.** (a) J–V curves, (b) L–V curves, (c) current efficiency curves, and (d) EQE curves of OLED devices incorporating GPF: SIS blend films with various SIS concentrations.


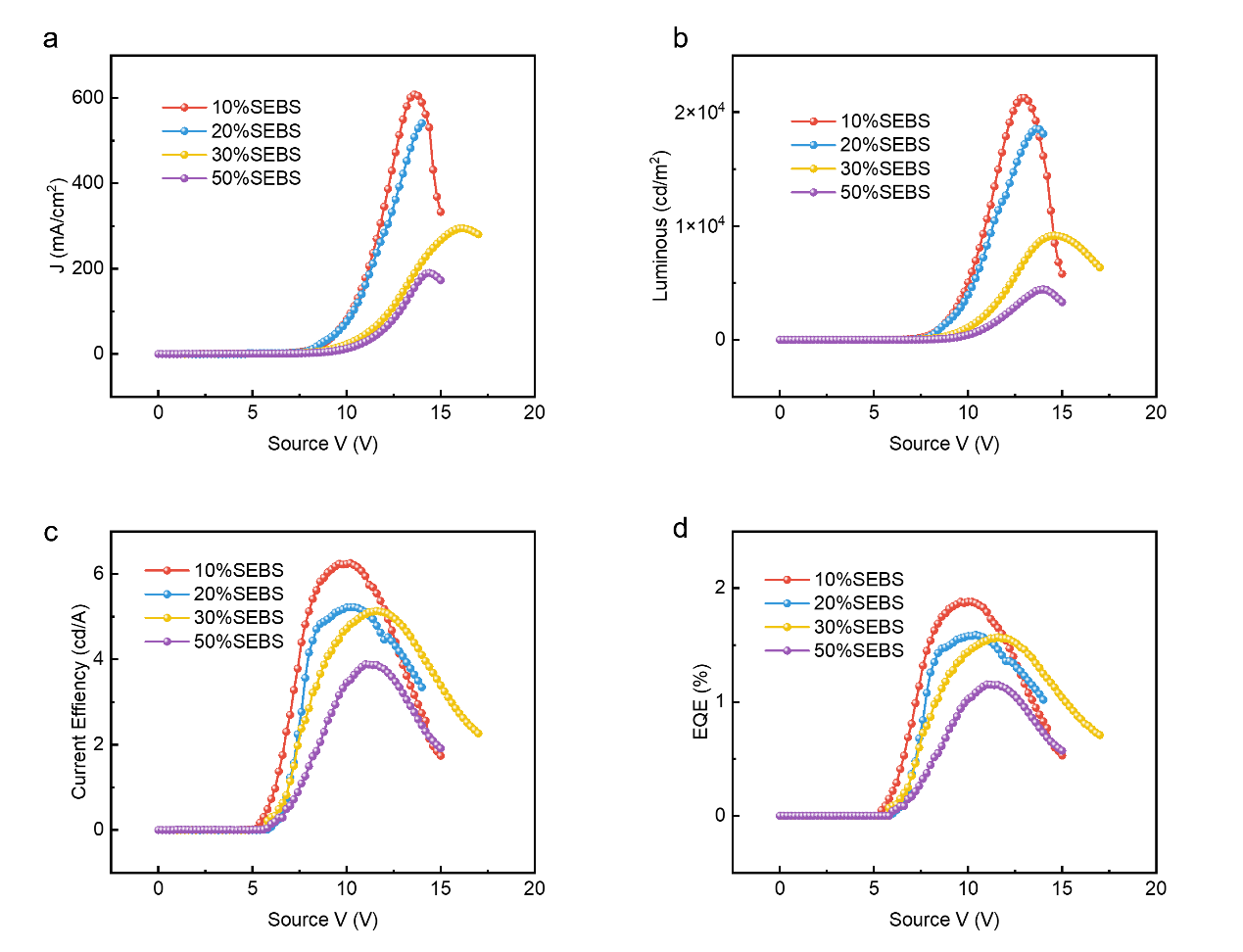


**Supplementary Fig 19.** (a) J–V curves, (b) L–V curves, (c) current efficiency curves, and (d) EQE curves of OLED devices incorporating GPF: SEBS blend films with various SEBS concentrations.


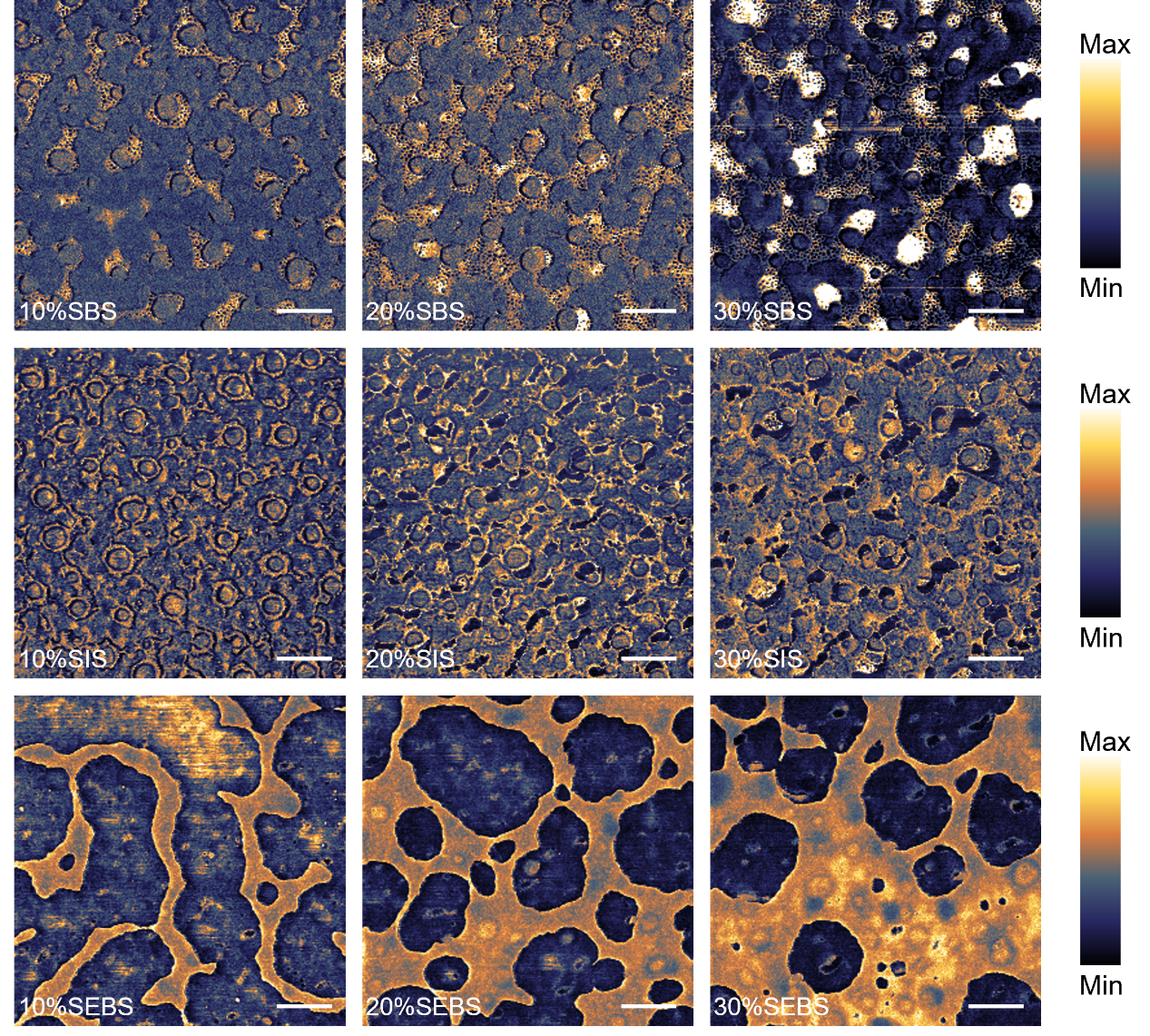


**Supplementary Fig 20.**  PiFM images of GPF films blended with varying concentrations (10%, 20%, and 30% by weight) of SBS, SIS, and SEBS. Scale bar is 500 nm.


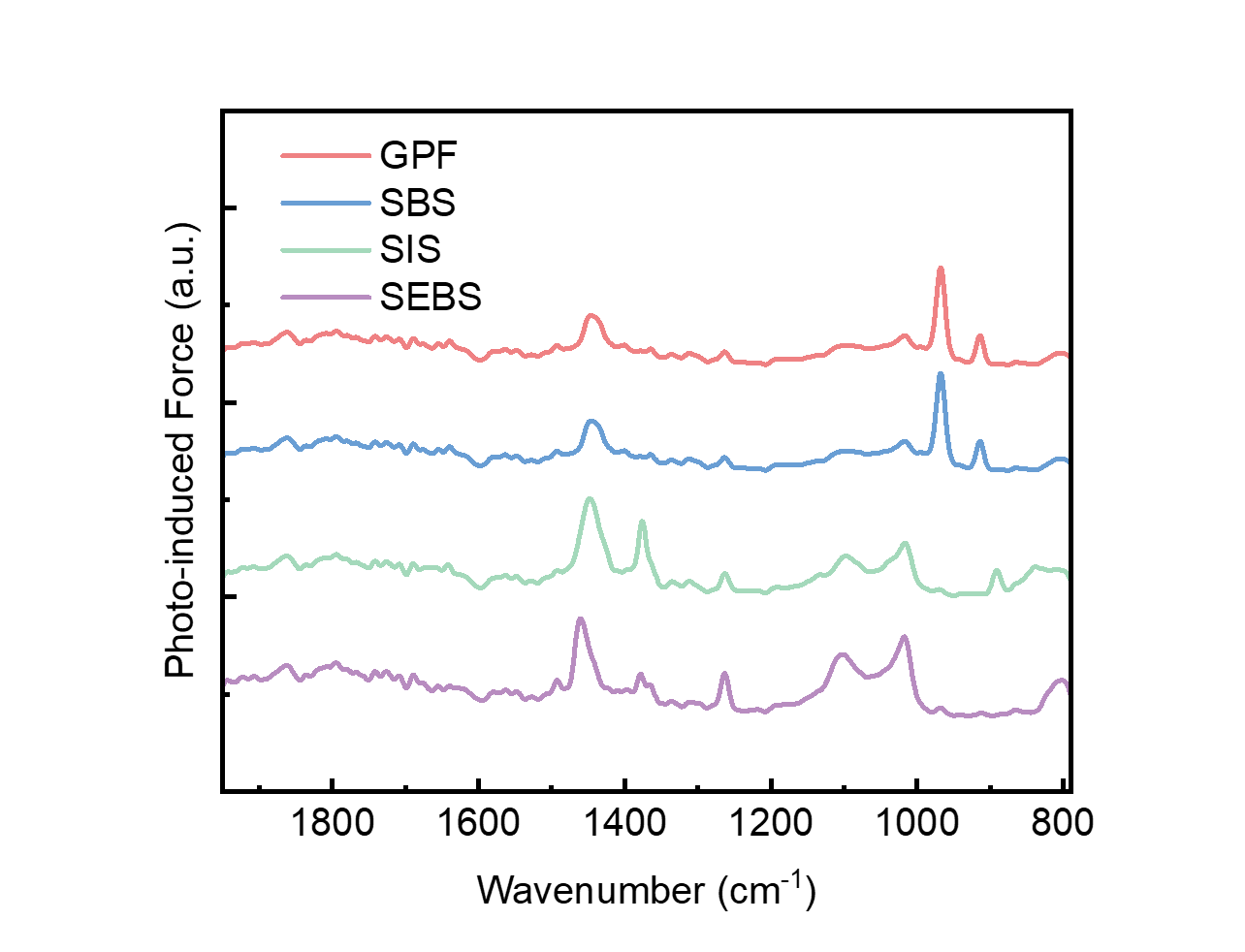


**Supplementary Fig 21.** Photo-induced Force Microscopy (PiFM) spectra of pristine GPF, SBS, SIS, and SEBS materials


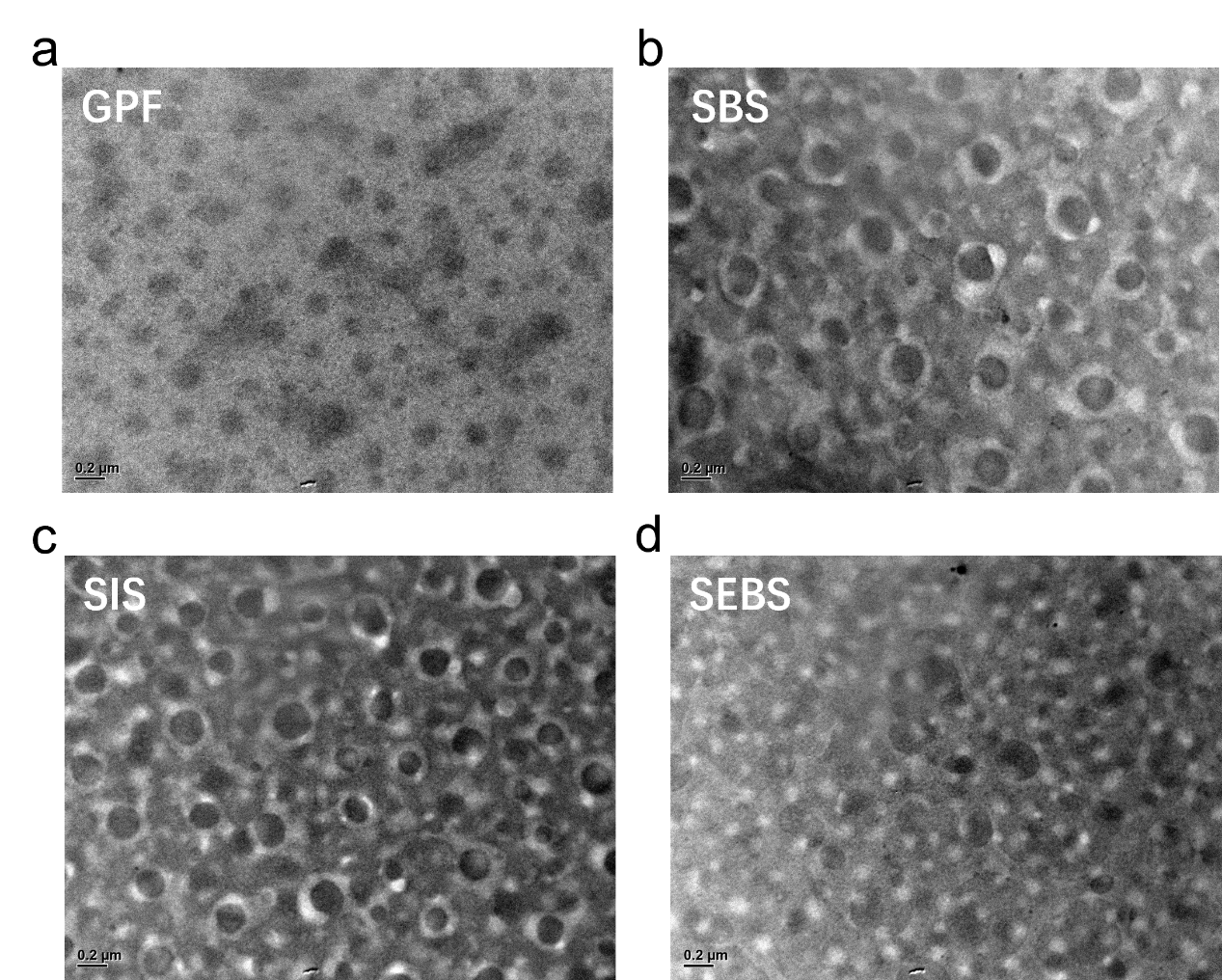


**Supplementary Fig 22.** TEM images of (a) pristine GPF film, and GPF films blended with (b) 10% SBS, (c) 10% SIS, and (d) 10% SEBS.


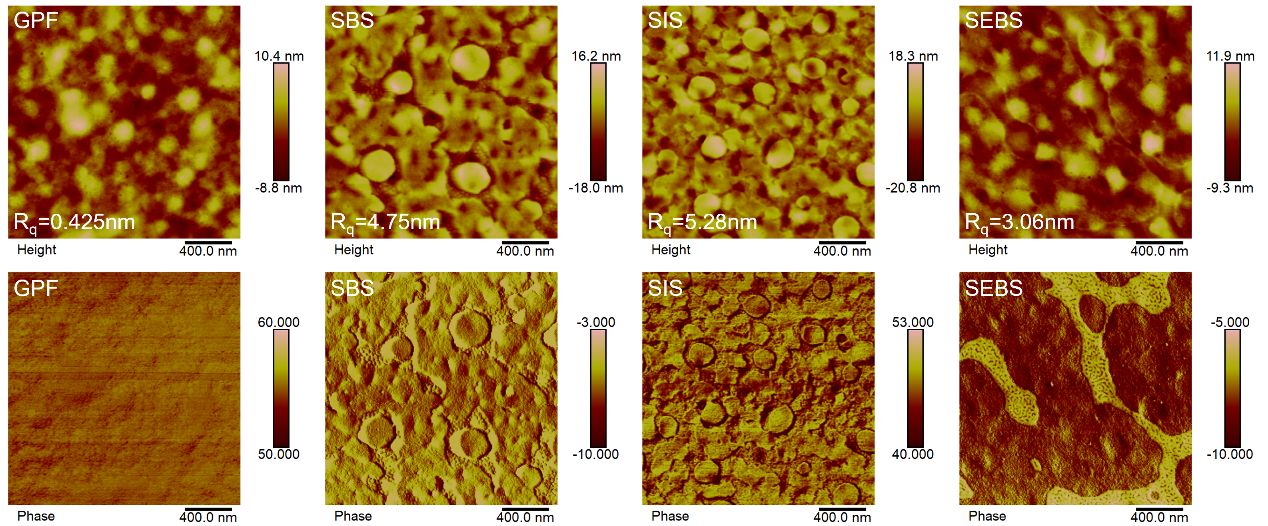


**Supplementary Fig 23**. AFM images of (a) pristine GPF film, and GPF films blended with (b) 10% SBS, (c) 10% SIS, and (d) 10% SEBS.


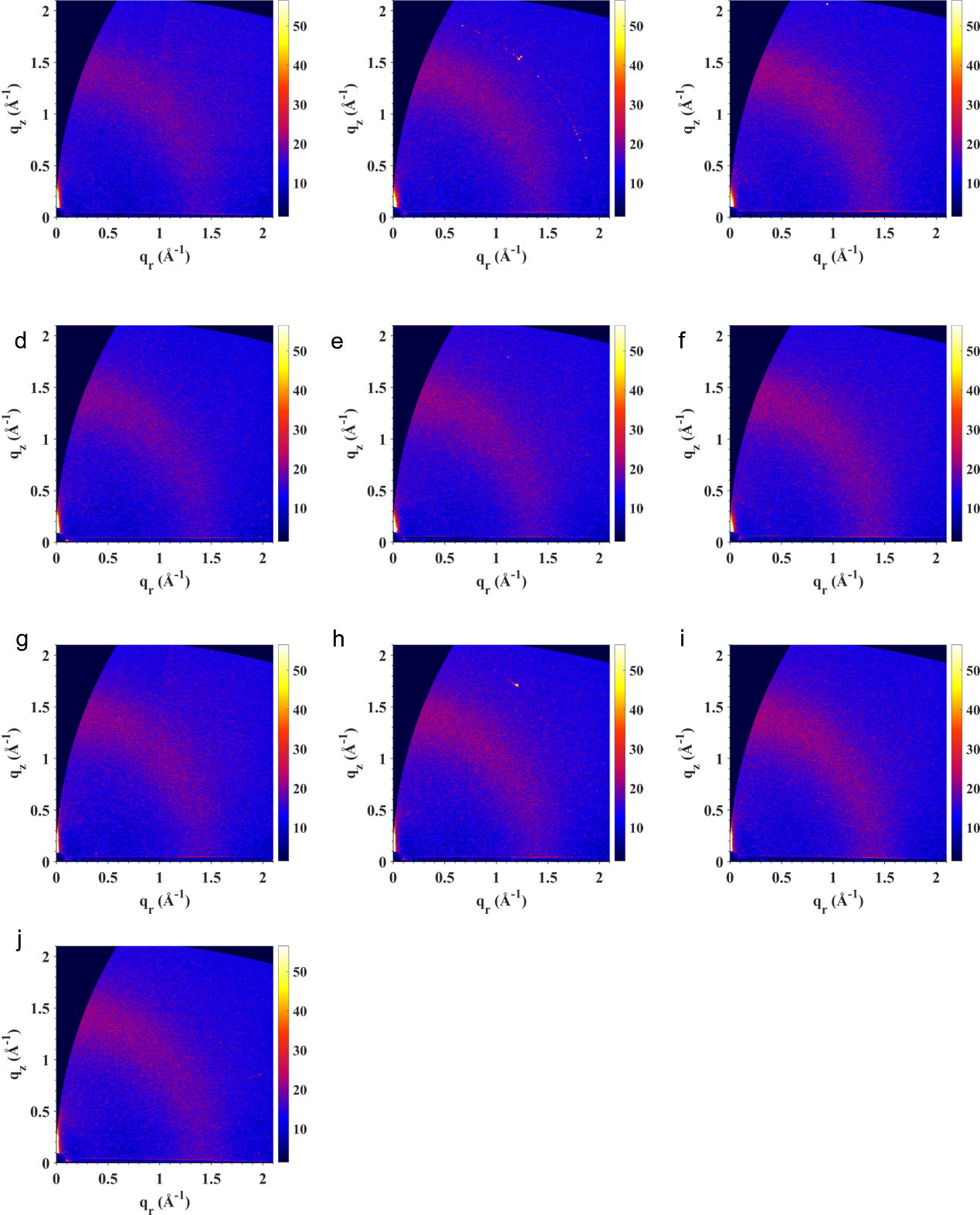


**Supplementary Fig 24.** Two-dimensional GIWAXS patterns of GPF films doped with varying concentrations of elastomers: (a–c) 10%, 20%, and 30% SBS; (d–f) 10%, 20%, and 30% SIS; (g–i) 10%, 20%, and 30% SEBS: (j) Pristine GPF.


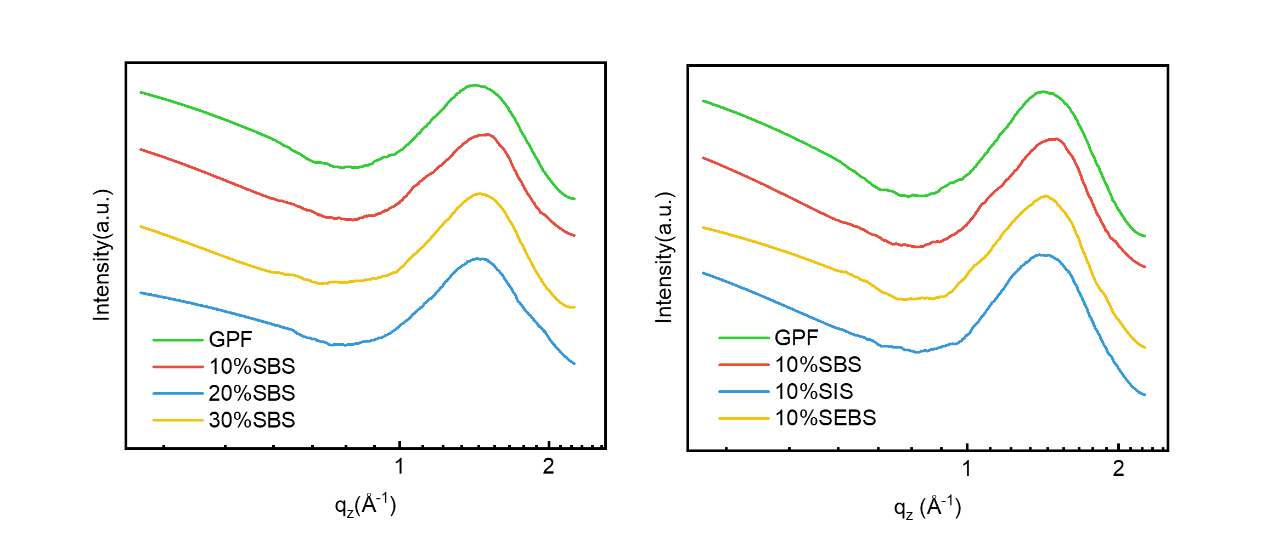
 **Supplementary Fig 25.** Out-of-plane GIWAXS line profiles of pristine GPF and GPF blended with with varying amounts of elastomers.


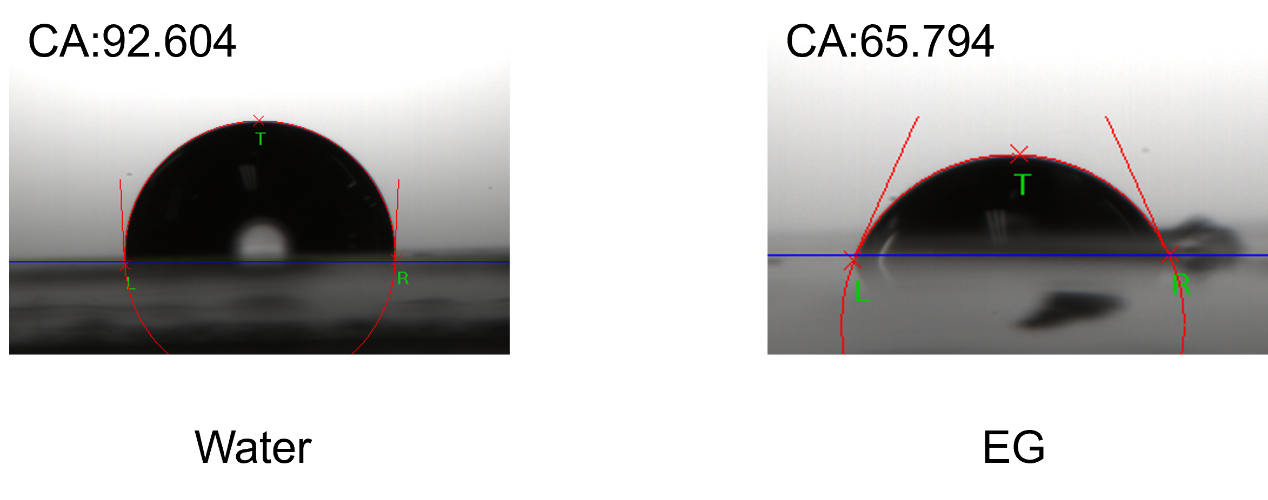


**Supplementary Fig 26**. Optical images showing the contact angles of SY film.


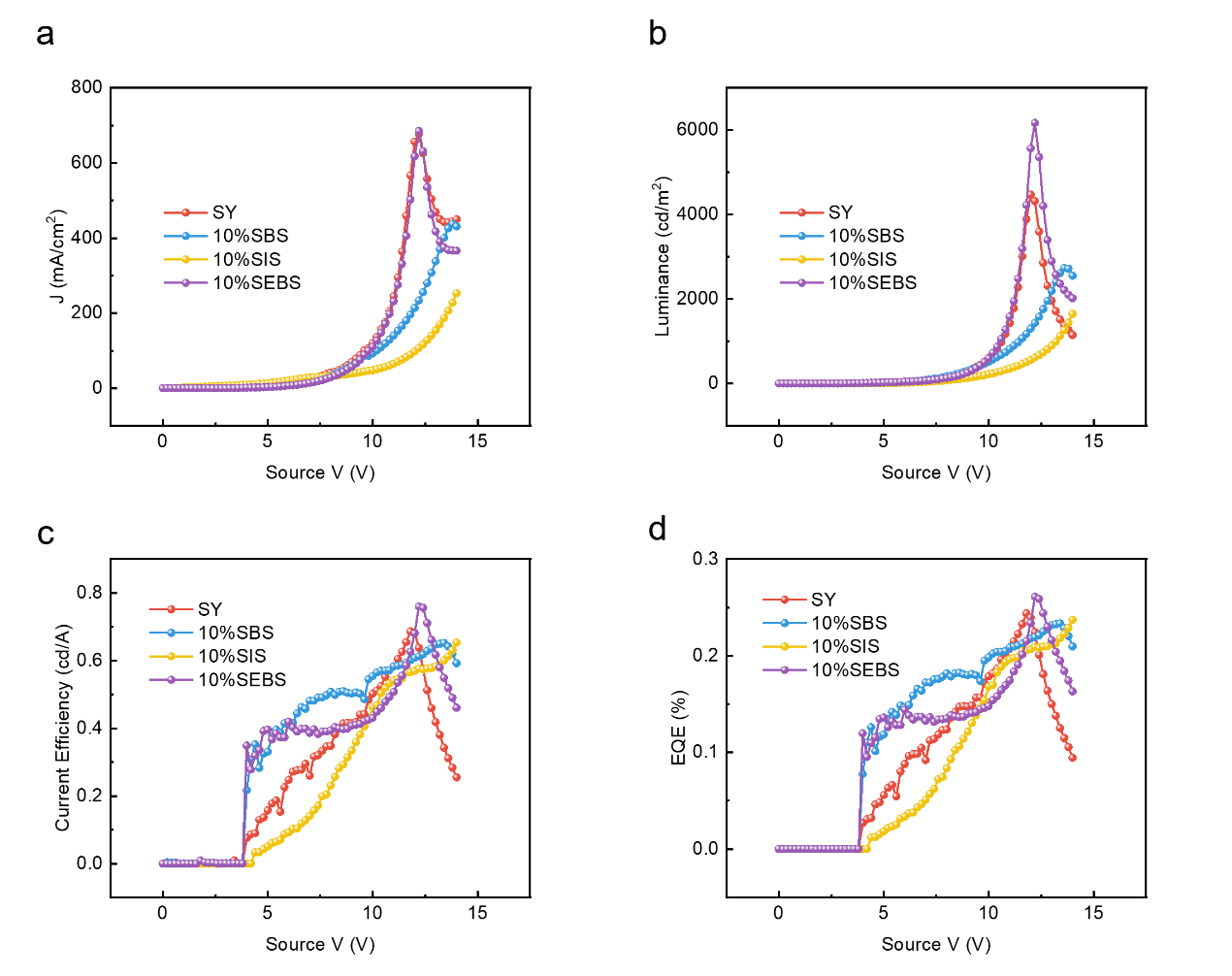


**Supplementary Fig 27.** (a) J–V curves, (b) L–V curves, (c) current efficiency curves, and (d) EQE curves for OLED devices incorporating SY blended with each of the three elastomers.


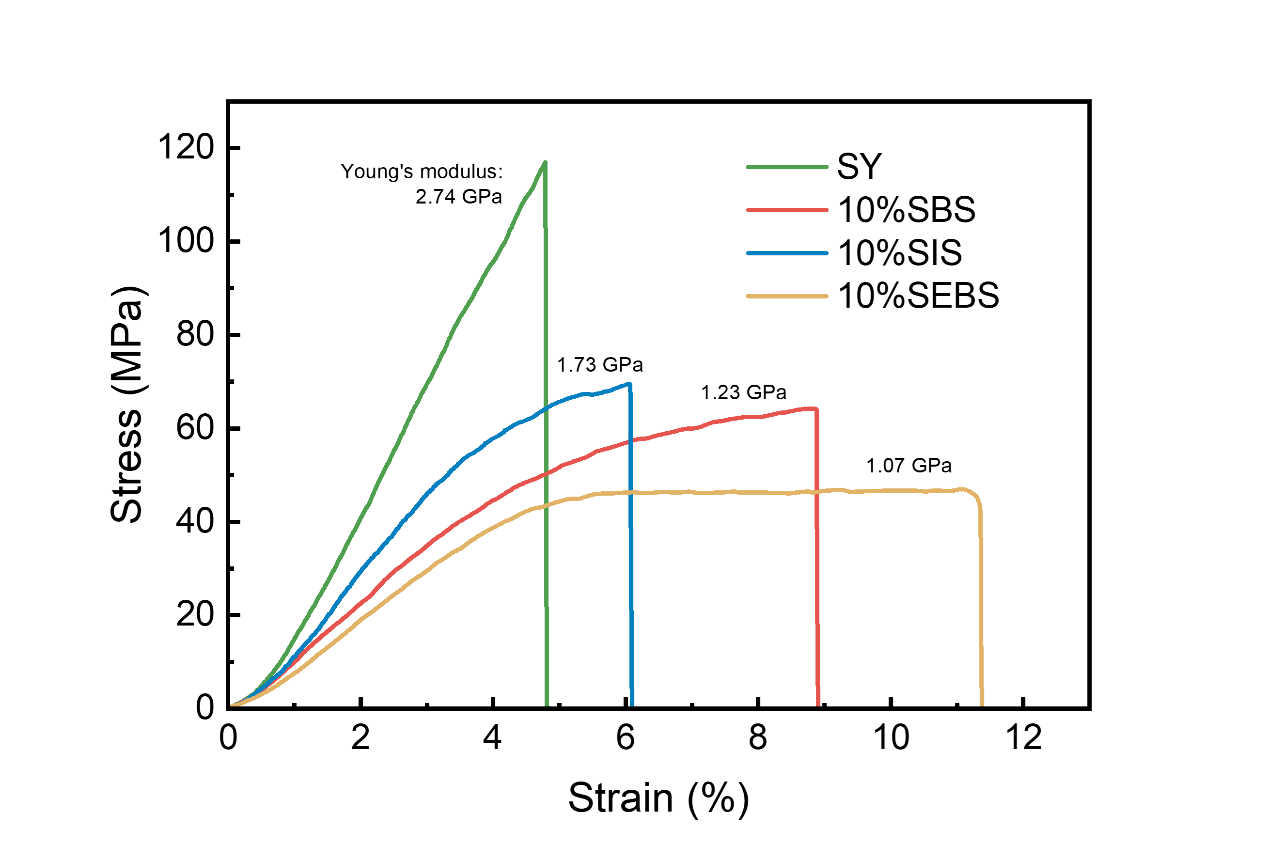


**Supplementary Fig 28.** Tensile stress curves for the film-on-water (FOW) configuration, comparing pristine SY and SY blend films with SBS, SIS, and SEBS.


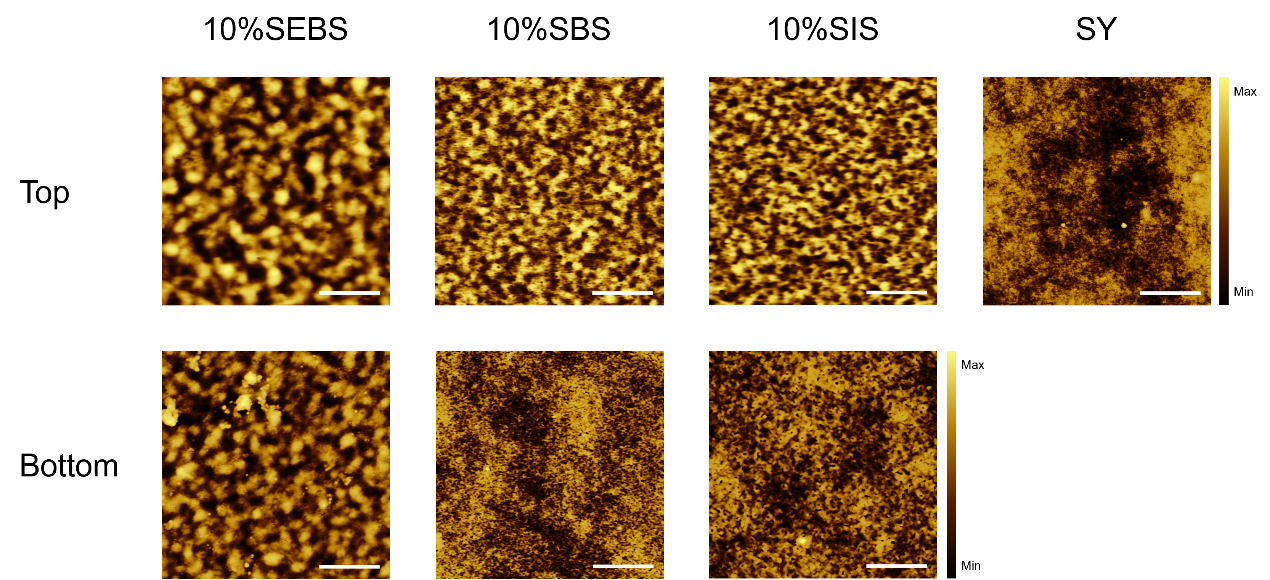


**Supplementary Fig 29.** AFM height images of pristine SY film and the top and bottom surfaces for SY films blended with 10% SEBS, 10% SBS, and 10% SIS. Scar bar: 1 μm.

*
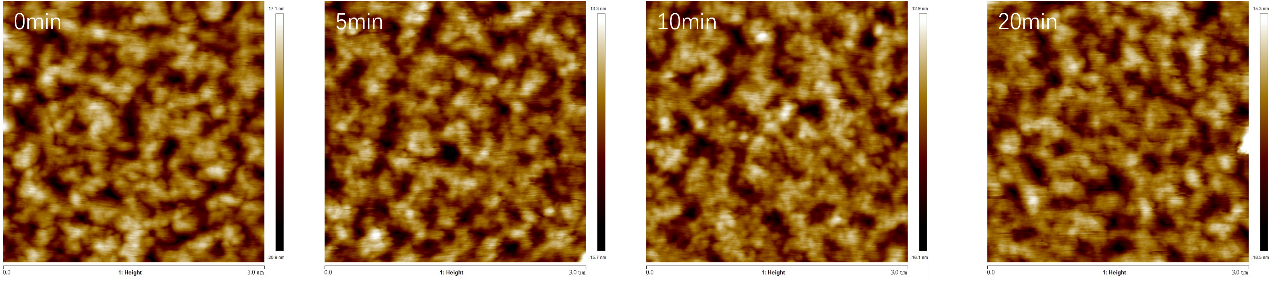
* **Supplementary Fig 30.** AFM topography images for PH1000 films with different time immersing in DI-water.


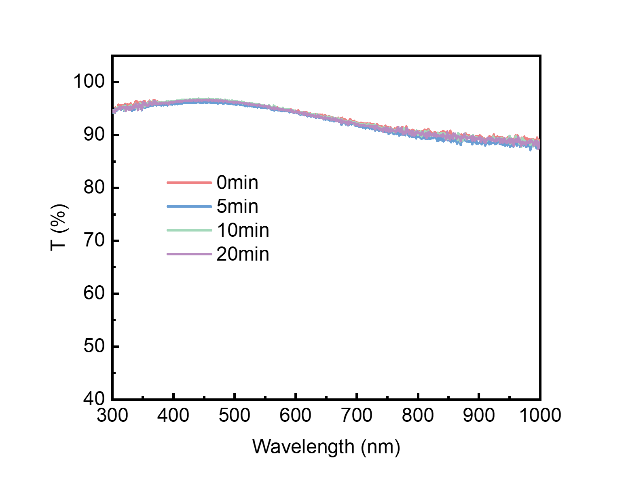


**Supplementary Fig 31.** Transmittance for PH1000 films with different time immersing in DI-water.


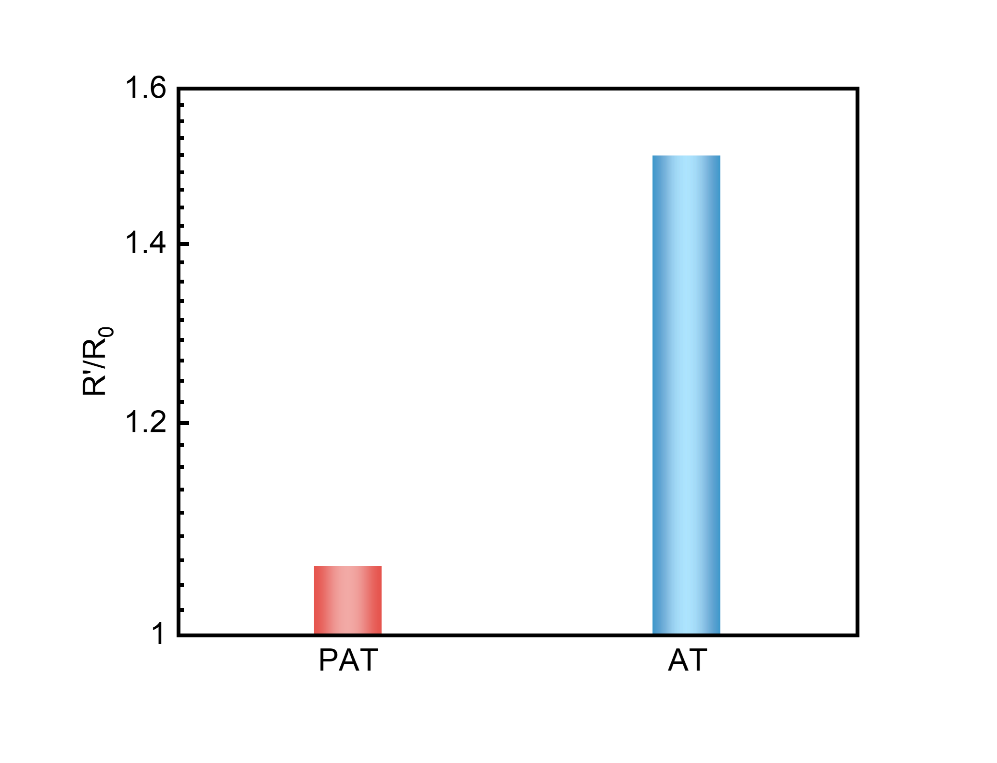


**Supplementary Fig 32.** Resistance changes before and after peeling-off for PAT and AT.


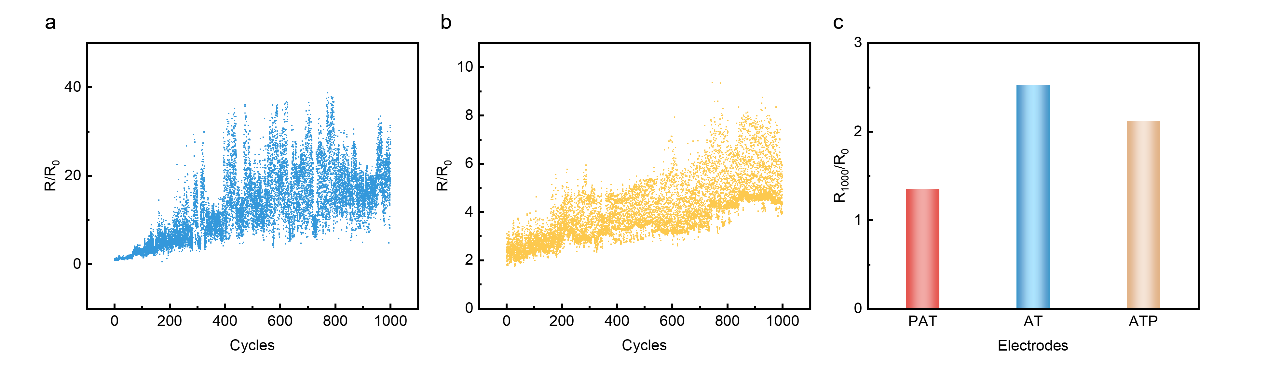


**Supplementary Fig 33.** Long-term mechanical stability under continuously stretching with a tensile strain of 20%, a) AT, b) ATP. (c) Relative resistance changes after 1000 stretching cycles.


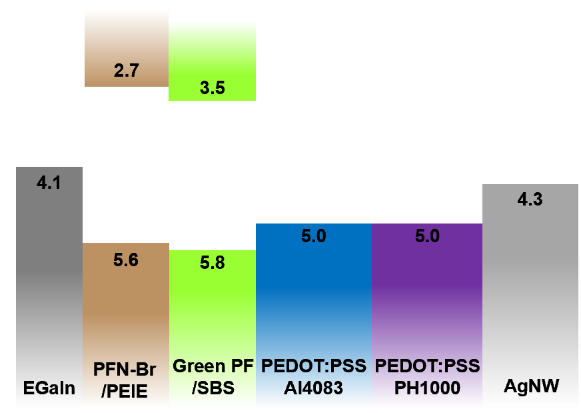


**Supplementary Fig 34.** Energy-level alignment diagram for the ISOLED.


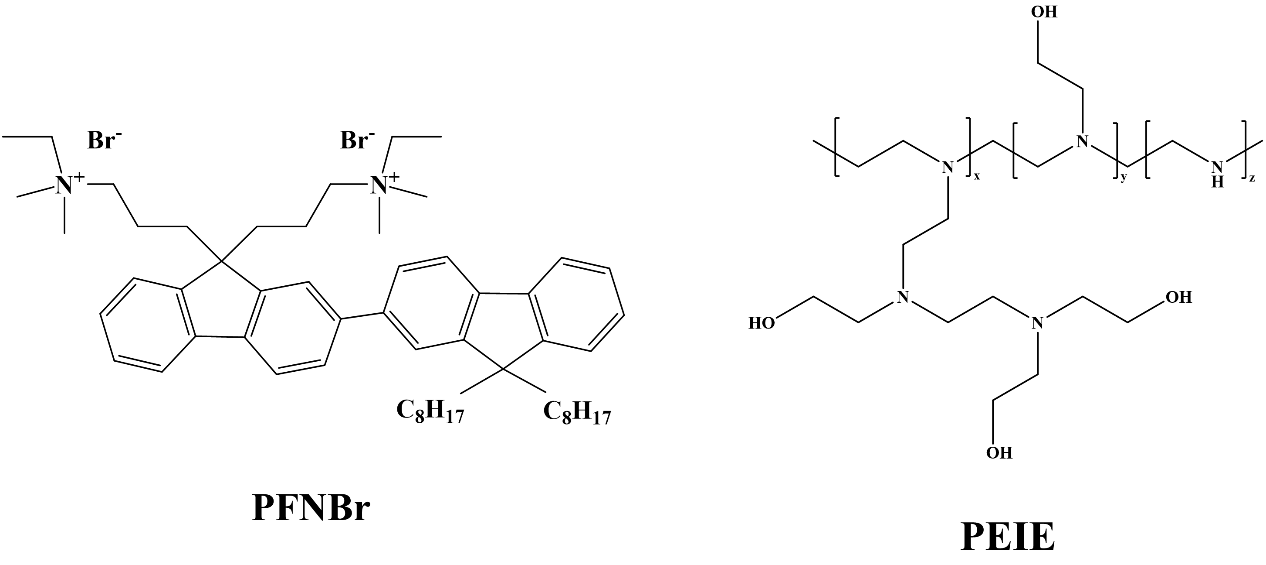


**Supplementary Fig 35**. Chemical structures of PFN-Br and PEIE.


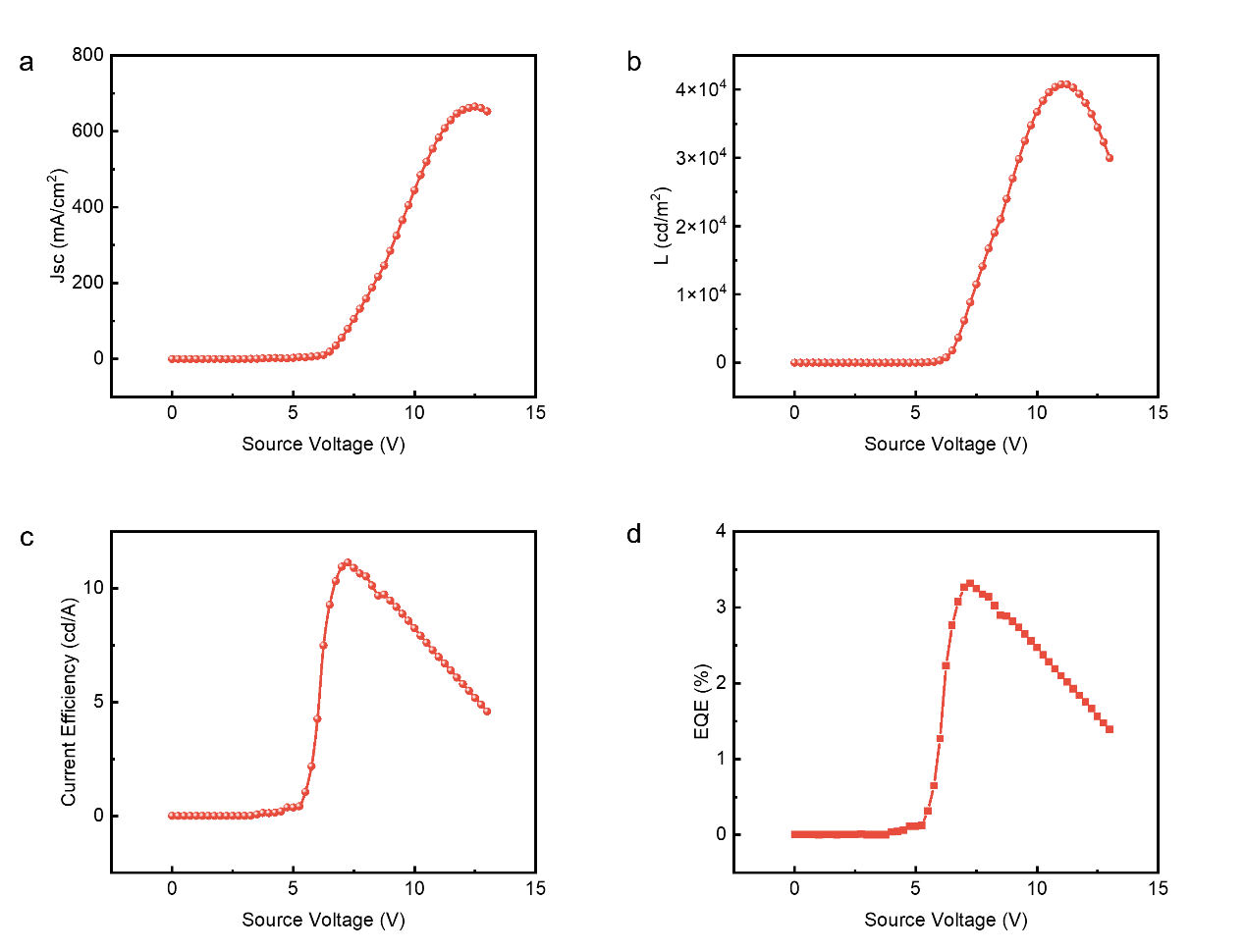


**Supplementary Fig 36.** (a) J–V curves, (b) L–V curves, (c) current efficiency curves, and (d) EQE curves of rigid OLED devices employing EGaIn as the top electrode.


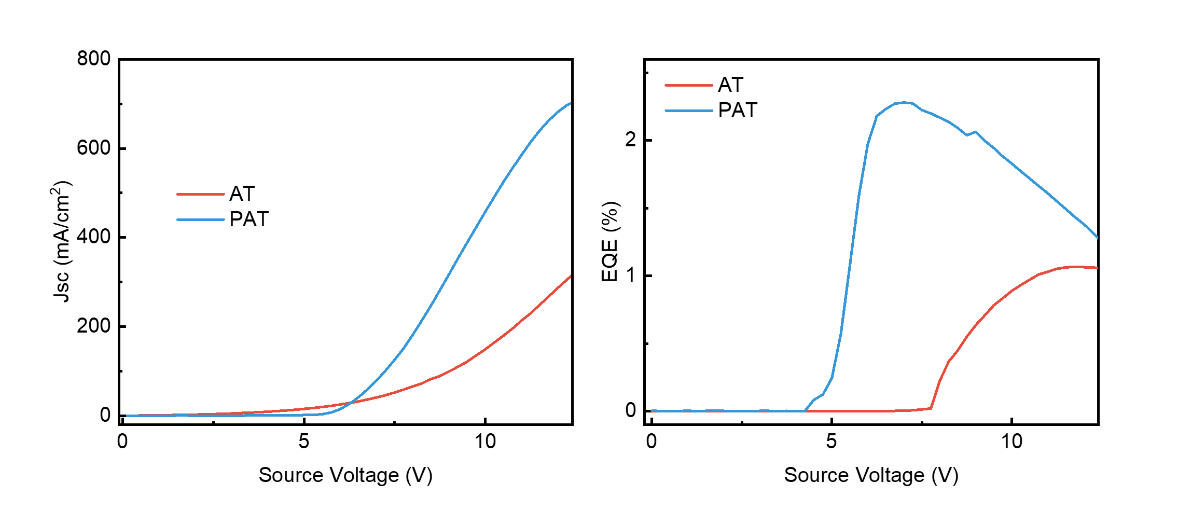


**Supplementary Fig 37.** JV curve and EQE versus voltage curves for *is*-OLEDs using two stretchable electrodes (ATP, PAT).


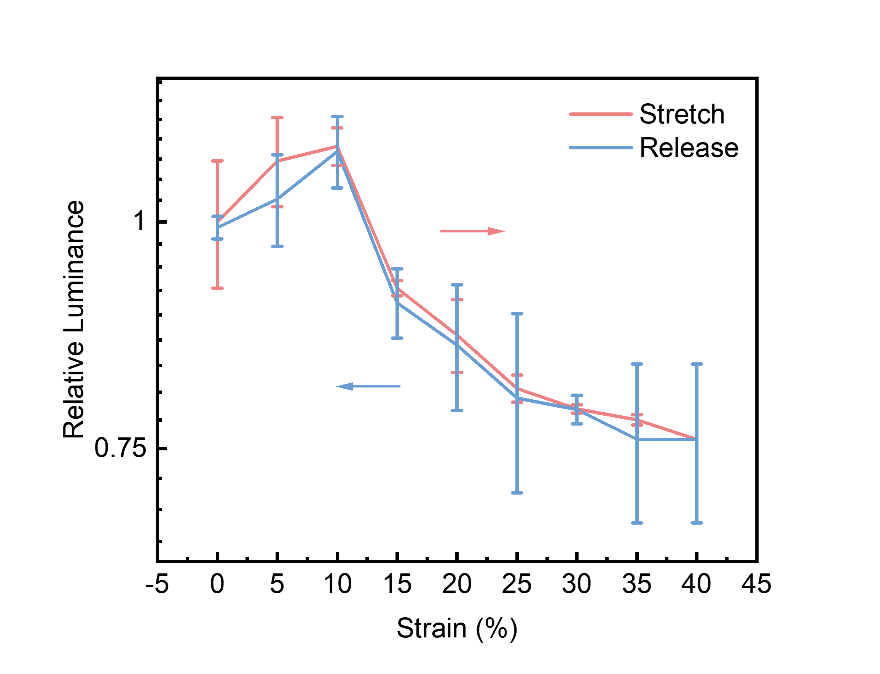


**Supplementary Fig 38.** Relative luminance of the stretchable OLED device during a stretching-releasing cycle. The data was collected from 3 independent devices.


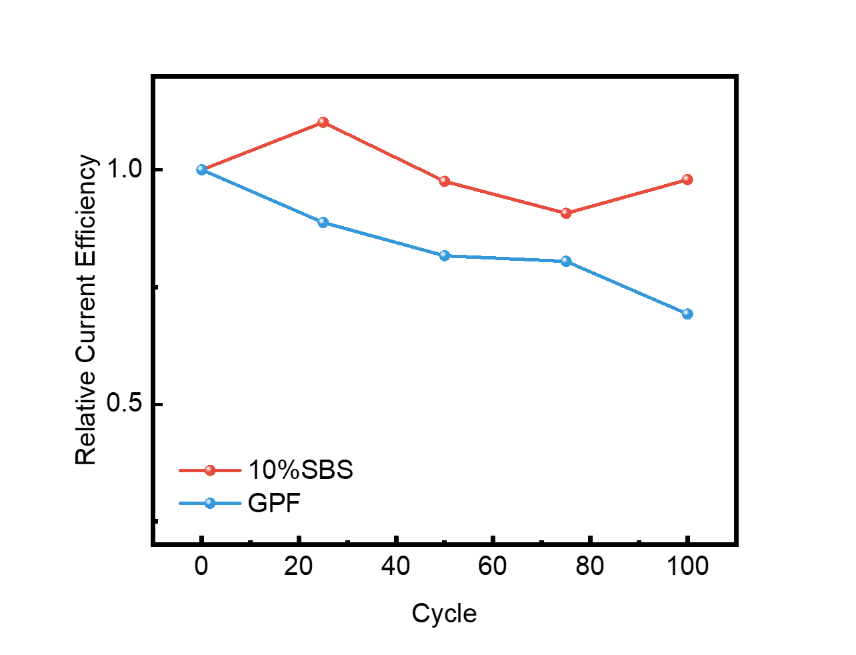


**Supplementary Fig 39.** Relative current efficiency changes under 100 repeated stretching cycles (15% strain) for the ISOLED based on a GPF: 10%SBS blended film, compared to a stretchable OLED using pristine GPF.


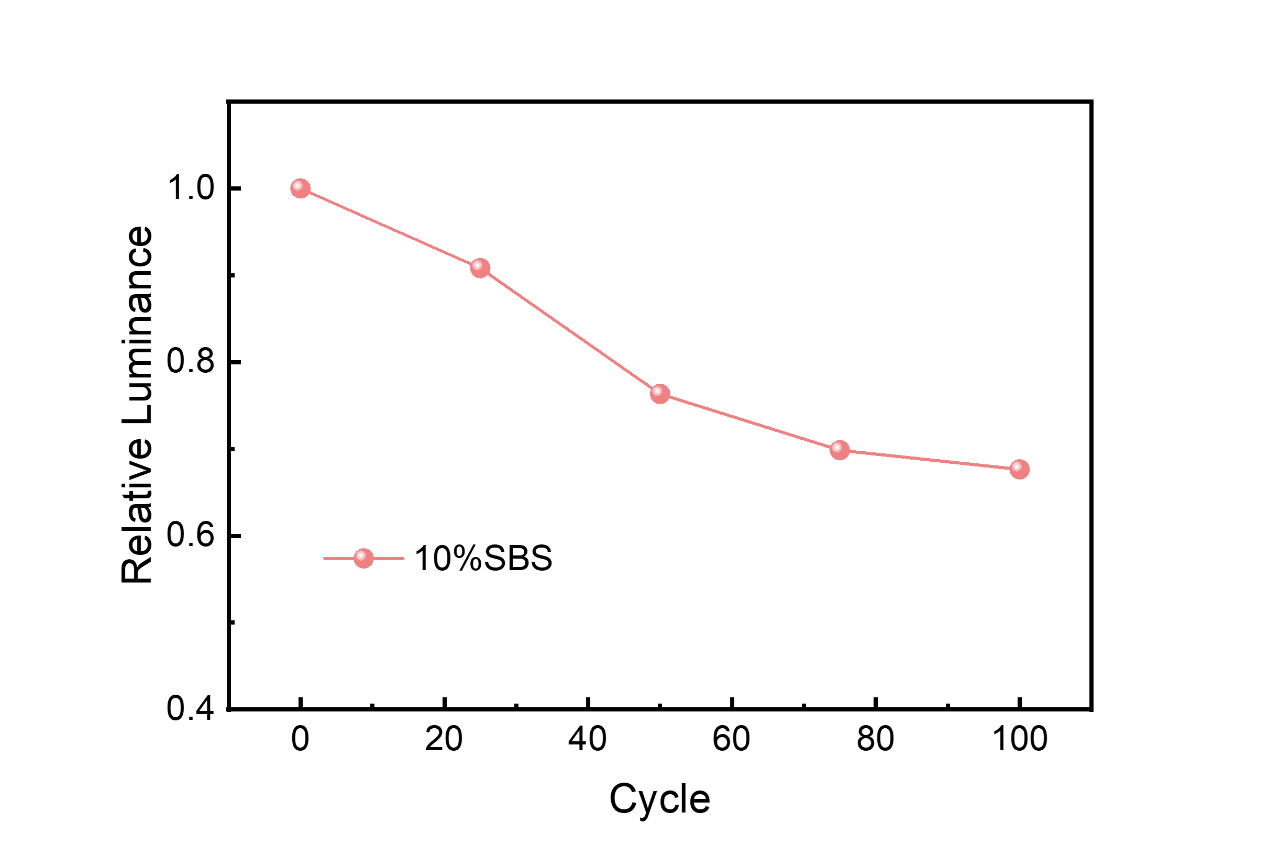


**Supplementary Fig 40.** Operational stability of the is-OLED based on the GPF:10%SBS blended film under 40% strain.


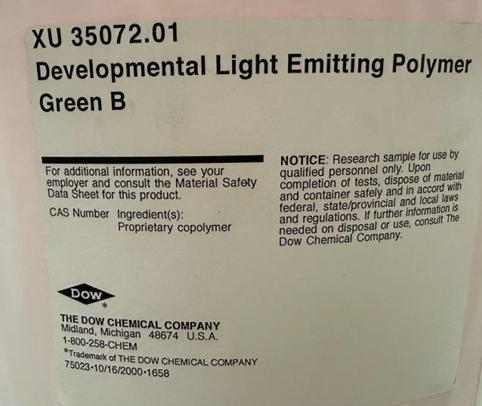

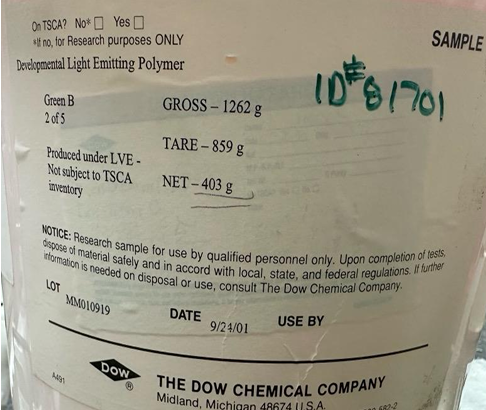


**Supplementary Fig 41.** Detailed information about the GPF material.

**Supplementary Table 1**. Surface Energies and Interaction Parameters for GPF and Three Elastomers.

|  | Contact Angle (deg) | |  | Surface Energy [mN m^-1^] | | |  |
| --- | --- | --- | --- | --- | --- | --- | --- |
|  | Water | Ethylene Glycol |  | γ_tot_ | γ_d_ | γ_p_ | Interaction Parameter(χ) |
| GPF | 95.56 | 68.36 |  | 27.14 | 25.44 | 1.70 | - |
| SBS | 98.64 | 70.72 |  | 27.41 | 26.47 | 0.94 | χ_GPF-SBS_: 0.00066K |
| SEBS | 104.18 | 72.29 |  | 32.84 | 32.83 | 0.01 | χ_GPF-SEBS_: 0.27114K |
| SIS | 99.47 | 73.22 |  | 24.93 | 23.79 | 1.15 | χ_GPF-SIS_: 0.04690K |
| SY | 92.25 | 65.72 |  | 36.73 | 35.75 | 0.98 | - |
| PEDOT:PSS |  |  |  | >40 |  |  |  |

“K” is the proportionality constant, γ_tot_ is the total surface energy, γ_d_ is the dispersive component, and γ_p_ is the polar component of the surface energies.

**Supplementary Table 2**. PL Lifetime Fitting Parameters and Calculated Lifetimes for Different Materials and Doping Ratios.

|  | GPF | 10%  SBS | 20%  SBS | 30%  SBS | 10%  SEBS | 20%  SEBS | 30%  SEBS | 10%  SIS | 20%  SIS | 30%  SIS |
| --- | --- | --- | --- | --- | --- | --- | --- | --- | --- | --- |
| A_1_ | 0.9319 | 0.5937 | 0.5437 | 0.6824 | 0.9915 | 0.9598 | 0.9336 | 0.7264 | 0.7645 | 0.8520 |
| t_1_ | 1.9031 | 1.1057 | 1.0231 | 1.0582 | 2.1005 | 1.9373 | 1.7721 | 1.4017 | 1.5148 | 1.6794 |
| A_2_ | 0.0383 | 0.3561 | 0.3713 | 0.3055 | 0.0195 | 0.0280 | 0.0569 | 0.2557 | 0.1830 | 0.1302 |
| t_2_ | 6.0919 | 2.3414 | 2.2857 | 2.2380 | 10.3623 | 6.5006 | 4.7823 | 2.8009 | 3.0746 | 3.5085 |
| t_ave_ | 1.1723 | 1.7083 | 1.7678 | 1.5738 | 1.3505 | 1.0493 | 1.1601 | 1.5753 | 1.4496 | 1.3003 |

A_1_: Amplitude of the fast decay component. t_1_: Time constant of the fast decay. A_2_: Amplitude of the slow decay component. t_2_: Time constant of the slow decay. t_ave_: Average lifetime calculated from the weighted contributions of both decay components.

**Supplementary Table 3**. Maximum Luminance and Current Efficiency of GPF with Varying Elastomer Doping Concentrations.

|  | Luminance (cd m^-2^) | Current Efficiency (cd A^-1^) | EQE(%) | |
| --- | --- | --- | --- | --- |
| GPF | 34239 | 7.12 | | 2.05 |
| 10%SBS | 39981 | 7.52 | | 2.16 |
| 20%SBS | 23259 | 7.28 | | 2.13 |
| 30%SBS | 12125 | 5.99 | | 1.82 |
| 50%SBS | 6070 | 5.92 | | 1.75 |
| 10%SIS | 29395 | 6.39 | | 1.92 |
| 20%SIS | 19529 | 6.09 | | 1.8 |
| 30%SIS | 9871 | 5.69 | | 1.69 |
| 50%SIS | 5303 | 5.67 | | 1.68 |
| 10%SEBS | 21281 | 6.24 | | 1.88 |
| 20%SEBS | 18537 | 5.22 | | 1.59 |
| 30%SEBS | 9167 | 5.14 | | 1.52 |
| 50%SEBS | 4430 | 3.88 | | 1.15 |

**Supplementary Table 4.** Maximum Luminance and Current Efficiency of SY with three Elastomer Doping.

|  | Luminance (cd m^-2^) | Current Efficiency (cd A^-1^) | EQE(%) |
| --- | --- | --- | --- |
| SY | 4473 | 0.69 | 0.24 |
| 10%SIS | 2731 | 0.75 | 0.27 |
| 10%SBS | 2735 | 0.65 | 0.24 |
| 10%SEBS | 5382 | 0.76 | 0.26 |

**Supplementary Table 5.** Performance Comparison of 10% SBS-Doped GPF with Different Electrodes Configurations: Luminance, EQE, and Current Efficiency.

|  | Luminance (cd m^-2^) | Current Efficiency (cd A^-1^) | EQE(%) | V _turn-on_(V) |
| --- | --- | --- | --- | --- |
| ATP-EGaIn | 12489 | 3.65 | 1.07 | 5.9 |
| PAT-EGaIn | 33443 | 7.99 | 2.28 | 4.4 |
| ITO-Ag | 37541 | 10.67 | 3.12 | 4.9 |
| ITO-EGaIn | 40764 | 11.14 | 3.32 | 4.7 |

**Supplementary Table 6.** Comparison of intrinsically stretchable light-emitting-diode device performance.

| Device structure | Max Stretchability | Max Luminance (cd m^-2^) | Max Current Efficiency  (cd A^-1^) | Max EQE (%) | REF |
| --- | --- | --- | --- | --- | --- |
| TPU/PEDOT:PSS(PH1000)/  AgNW/PEDOT:PSS(Al 4083)/GPF:SBS/PFNBr:PEIE/EGaIn | 120 | 33443 | 7.99 | 2.3 | This work |
| PDMS/AgNW/PEDOT:PSS:TX/SY:TX/ZnO/d-EIE/AgNW/ZnO/d-PEIE | 80 | 4400 | 1.60 | - | FL^1^ |
| Au (30 nm)/Au interlayer (3 nm)/PEDOT:PSS-TX (80 nm)/L-SY-PPV-PAN (5:5, 70 nm)/PMMA(1 nm)/Zn-PEIE-pBphen-TR (15:1:6:1, 65 nm)/Ag interlayer (2 nm)/AgNWs/PDMS | 30 | 3780 | 2.35 | 0.69 | FL^2^ |
| TCSE/PEDOT:PSS/ SY:PEO:KCF_3_SO_3_/PEI/TCSE | 20 | 2185 | 20.30 | - | FL^3^ |
| PVDFHFP/PEDOT:PSS:PR/PEDOT:PSS:TritonX/TFB:PU/SY:PU/PFNBr:PEIE/PEDOT:PSS:PR/P VDF-HFP | 100 | 7450 | 5.3 | - | FL^4^ |
| PEDOT:PSS (PH 1000)/ PEDOT:PSS (Al 4083)/ EMLs/TPBi/LiF/Al | 54 | 3274 | 5.32 | 2.08 | FL^5^ |
| TCSE/PEDOT:PSS/SY:TX/Crown-CPE/PEI/TCSE | 30 | 1754 | 3.14 | - | FL^6^ |
| ITO/PEDOT:PSS/PFO:SEBS/ZnO/Al* | 100 | 1000 | 0.60 | - | FL^7^ |
| ITO/PEDOT:PSS/PVK/Z1:F8BT/TPBi/Al/LiF* | 25 | 2000 | 2.00 | 0.35 | FL^8^ |
| PDMS AgNW/PEDOT:PSS:TX/  SY:TX/PFNBr:PEIE:TX/Ag/d-PEIE | 70 | 3153 | 5.40 | - | FL^9^ |
| AgNW/PEDOT:PSS_PFI/ PDKCD/PEIE_PFNBr/AgNW | 60 | 2000 | 10.20 | 3.3 | **TADF**^10^ |
| UV-curable PUA/AgNW:PEDOT/TFB/Q_100_S_10_T_5_­/ZnO:PFNBr/AgNW:Ag:liquid metal/UV-curable PUA | 50 | 15170 | 5.0 | 1.2 | **QLED**^11^ |

* Note: These devices utilize an inherently stretchable light-emitting layer on a rigid electrode to determine efficiency.

**Supplementary Table 7.** Comparison of luminance retention of intrinsically stretchable light-emitting-diodes devices under cyclic stretching.

| Cycles | Strain (%) | L/L_0_(%) | Ref |
| --- | --- | --- | --- |
| 100 | 15 | 88 | This work |
| 100 | 40 | 68 | This work |
| 50 | 15 | 46 | 3 |
| 50 | 10 | 57 | 7 |
| 100 | 40 | 85 | 5 |
| 100 | 10 | ~100 | 9 |
| 300 | 40 | 80 | 10 |
| 50 | 50 | 80 | 2 |

Note: "Cycles" denotes the number of stretch-release cycles performed. "Strain" indicates the maximum tensile strain applied during the cycles. "L/L_0_" represents the percentage of luminance retained after the specified number of cycles compared to the initial luminance.

**Supplementary Table 8.** Surface roughness (Rq) and transmittance at 550 nm (T_550_%) of the PH1000 layer after various immersion times in deionized water.

| Minutes | Rq | T_550_(%) |
| --- | --- | --- |
| 0 | 5.65 | 95.50 |
| 5 | 4.27 | 95.19 |
| 10 | 4.19 | 95.55 |
| 20 | 4.86 | 95.43 |

**References**

1 Kim, J. H. & Park, J. W. Intrinsically stretchable organic light-emitting diodes. *Science Advances* **7**, eabd9715 (2021).

2 Liu, Y. W. *et al.* A Self-Assembled 3D Penetrating Nanonetwork for High-Performance Intrinsically Stretchable Polymer Light-Emitting Diodes. *Advanced Materials* **34**, e2201844 (2022).

3 Zhou, H. Y. *et al.* Graphene-Based Intrinsically Stretchable 2D-Contact Electrodes for Highly Efficient Organic Light-Emitting Diodes. *Advanced Materials* **34**, 2203040 (2022).

4 Zhang, Z. T. *et al.* High-brightness all-polymer stretchable LED with charge-trapping dilution. *Nature* **603**, 624–630 (2022).

5 Li, X. C. *et al.* Intrinsically Stretchable Electroluminescent Elastomers with Self-Confinement Effect for Highly Efficient Non-Blended Stretchable OLEDs. *Angewandte Chemie International Edition* **62**, e202213749 (2023).

6 Han, S. J., Zhou, H. Y., Kwon, H., Woo, S. J. & Lee, T. W. Achieving Low-Voltage Operation of Intrinsically-Stretchable Organic Light-Emitting Diodes. *Advanced Functional Materials* **33**, 2211150 (2023).

7 Jeong, M. W. *et al.* Intrinsically stretchable three primary light-emitting films enabled by elastomer blend for polymer light-emitting diodes. *Science Advances* **9**, eadh1504 (2023).

8 Zhuo, Z. Q. *et al.* Intrinsically stretchable fully π-conjugated polymer film via fluid conjugated molecular external-plasticizing for flexible light-emitting diodes. *Nature Communications* **15**, 7990 (2024).

9 Oh, J. H., Jeon, K. H. & Park, J. W. Intrinsically stretchable OLEDs with a designed morphology-sustainable layer and stretchable metal cathode. *npj Flexible Electronics* **8**, 43 (2024).

10 Liu, W. *et al.* High-efficiency stretchable light-emitting polymers from thermally activated delayed fluorescence. *Nature Materials* **22**, 737–745 (2023).

11 Kim, D. C. *et al.* Intrinsically stretchable quantum dot light-emitting diodes. *Nature Electronics* **7**, 365–374 (2024).
